# Extracellular vesicles released by fibroblasts undergoing H-Ras induced senescence show changes in lipid profile

**DOI:** 10.1371/journal.pone.0188840

**Published:** 2017-11-28

**Authors:** Sandra Buratta, Lorena Urbanelli, Krizia Sagini, Stefano Giovagnoli, Silvia Caponi, Daniele Fioretto, Nico Mitro, Donatella Caruso, Carla Emiliani

**Affiliations:** 1 Department of Chemistry, Biology and Biotechnology, University of Perugia, Perugia, Italy; 2 Department of Pharmaceutical Sciences, University of Perugia, Perugia, Italy; 3 Istituto Officina dei Materiali del CNR (CNR-IOM) - Unità di Perugia, c/o Department of Physics and Geology, University of Perugia, Perugia, Italy; 4 Department of Physics and Geology, University of Perugia, Perugia, Italy; 5 CEMIN-Center of Excellence for Innovative Nanostructured Material, Perugia, Italy; 6 Department of Pharmacological and Biomolecular Sciences, University of Milan, Milan, Italy; Universita degli Studi di Torino, ITALY

## Abstract

Cells release extracellular vesicles (EVs) in their environment and cellular lipids play an important role in their formation, secretion and uptake. Besides, there is also evidence that EV transferred lipids impact on recipient’s cell signaling. Cellular senescence is characterized by a state of permanent proliferation arrest and represents a barrier towards the development of neoplastic lesions. A peculiar feature of senescence is the release of many soluble factors, the so-called Senescence-Associated Secretory Phenotype, which play a key role in triggering paracrine senescence signals. Recently, evidences have suggested that this phenotype includes not only soluble factors, but also EVs. To identify lipid signatures associated with H-Ras-induced senescence in EVs, we expressed active H-Ras (H-RasV12) in human fibroblasts and investigated how it affects EV release and lipid composition. An enrichment of hydroxylated sphingomyelin, lyso- and ether-linked phospholipids and specific H-Ras-induced senescence signatures, e.g. sphingomyelin, lysophosphatidic acid and sulfatides, were found in EVs compared to cells. Furthermore, H-RasV12 expression in fibroblasts was associated with higher levels of tetraspanins involved in vesicle formation.

## Introduction

Cells can exchange information through the release of small vesicular bodies called extracellular vesicles (EVs) [1–3]. All cell types investigated so far have been found to release EVs. It is now accepted that cells can release several types of EVs, characterized by heterogenous size, biogenesis and cargo [4]. EVs can originate either from the plasma membrane (microvesicles or ectosomes) or from the endosomal system (exosomes). Microvesicles show a wide size distribution, ranging from a few dozens of nanometers to a few micrometers, whereas vesicles originating from the internal budding of late endosomes have a size limited by their confinement within this compartment, i.e. usually between 30 and 150 nm [5,6].

By releasing EVs, cells spread in the extracellular space chemical signals, including proteins, nucleic acids and lipids. In the last few years EVs have been characterized mainly in their nucleic acid and protein content. Although lipids play a fundamental role in EV formation, secretion and uptake by target cells [7–10], the EV lipid content has been poorly investigated. As for proteins, the EV lipid composition is different from that of the releasing cell, but it is somehow related to the cell type. Likewise lipid rafts, EVs are often enriched in cholesterol, glycosphingolipids, sphingomyelin (SM), and saturated glycerophospholipids [10–12]. Recently, lipidomics of EVs isolated from melanoma cell lines has showed a great enrichment in SM, phosphatidylserine (PS), lysophosphatidylcholine (LPC) and lysophosphatidylethanolamine (LPE) with respect to cells [13]. Llorente and coworkers [14] demonstrated that EVs released from PC3 prostate cancer cells are highly enriched in glycosphingolipids, SM, cholesterol, and PS. Lydic and coworkers [15] observed increased levels of alkyl ether-containing glycerophospholipids in EVs secreted by LIM1215 colorectal cancer cell line with respect to cells. Overall, these studies showed that EVs possess highly ordered membranes, which is relevant for their stability in the extracellular environment and interaction with target cells [11]. The lipid content of EVs also represents a source of lipid mediators outside the cell that could trigger paracrine signals [16]. Nevertheless, the impact of EV lipids on the signal transduction of the target cell is poorly understood and further studies regarding the lipid composition of EVs in different physiological and pathological conditions are crucial.

Cellular senescence is a complex process characterized by a state of permanent proliferation arrest. It represents a safety mechanism allowing organisms to block the proliferation of damaged cells, which would otherwise proceed towards oncogenic transformation. Different stimuli can induce cellular senescence; in particular, DNA damage appears to be a key factor. Besides, oncogene activation has been also found to prompt cellular senescence, leading to the definition of peculiar senescence process termed Oncogene-Induced Senescence (OIS) [17].

One of the peculiar features of cellular senescence is the release in the extracellular environment of a number of soluble components, the so-called Senescence-Associated Secretory Phenotype (SASP). This includes cytokines, chemokines, growth factors and extracellular proteases, that facilitate the removal of senescent cells by phagocytic immune cells and the proliferation of neighbor tissue to promote repair [18]. In this regard, recent studies have suggested that EVs represent a new, poorly characterized component of SASP [19,20]. As a matter of fact, cellular senescence is associated with an increased EV release, and specifically one of the consequences of DNA damage is the secretion of EVs mediated by p53 activation [21,22].

In this work, we carried out a comprehensive lipidomic analysis of EVs released by human fibroblasts undergoing OIS. To gain a systematic overview, the lipid profile of EVs released from fibroblasts expressing constitutively active H-RasV12 and fibroblasts transfected with the empty vector as a control were compared. Besides, we also analyzed the lipid profile of their parental cells, i.e. H-RasV12 and control fibroblasts. This approach provided a comparison between the lipid profile of EVs in normal and senescence-associated conditions, which could allow the identification of potential biomarkers and metabolic alterations related to H-Ras-induced senescence.

## Materials and methods

### Reagents

Cell culture reagents, Lipofectamine LTX and blasticidin-S were from Life Technologies. All HPLC solvents were MS grade (Carlo Erba Reagents, Italia). Phospholipid standards: C13:0 lysophosphatidylcholines (lysoPC); C25:0 phosphatidylcholines (PC); C12:0 sphingomyelin (SM); 12:0–13:0 phosphatidylserine (PS); 12:0–13:0 phosphatidylinositol (PI); 12:0–13:0 phosphatidylglycerol (PG); 12:0–13:0 phosphatidic acid (PA); 12:0–13:0 phosphatidylethanolamine (PE); C12 ceramide (Cer); glucosyl (β) C12 ceramide (GCer); lactosyl (β) C12 ceramide (LacCer); C17 mono-sulfo galactosyl(β) ceramide (D18:1/17:0) were purchased from Avanti Polar Lipids. All other reagents were from Sigma-Aldrich.

### Cell culture and H-Ras expression

HuDe (human dermal fibroblasts) were purchased from the Istituto Zooprofilattico Sperimentale, Brescia, Italy as previously reported [17]. Cells were cultured in Dulbecco’s modified Eagle’s medium (DMEM) containing 10% (v/v) heat-inactivated fetal bovine serum (FBS), 2 mM glutamine, 100 U/mL penicillin, 100 mg/mL streptomycin and grown at 37°C in a 5% CO_2_. Cell viability was estimated by examining their ability to exclude trypan blue 0.1% (w/v) in 0.9% (w/v) NaCl. Cells were transiently transfected with the pcDNA6 plasmid encoding the constitutively active mutant H-RasV12, cloned as previously described [17], and with empty vector as control. Briefly, 1.8x10^5^ cells were seeded in 6-well plates and transfected with 1 μg/well of plasmid DNA using 3 μl/well of Lipofectamine LTX, according to manufacturer’s instructions; the day after cells were transferred into 100 mm plates and grown in the presence of 4 μg/ml blasticidin-S as selective agent for 5 to 7 days, then used for further analyses.

### Extracellular vesicles purification

HuDe fibroblasts transfected with H-RasV12 or with pcDNA6 were selected as described above. Before EVs recovery, cells were incubated for 72 hrs in serum free medium containing 4 μg/ml blasticidin-S to avoid any contamination by FBS lipoproteins that could affect EVs lipidomic analysis. Cells were counted in a haemocytometer and their viability was estimated as above. Medium was collected and underwent serial centrifugation steps to remove cells, cell debris and large EVs (300 × g, 10 min; 2,000 × g, 10 min), plus a filtration step at 0.22 μm with cellulose filter (Millipore), to enrich for small extracellular vesicles. EVs were isolated by polymer co-precipitation using Exoquick-TC^™^ precipitation method (System Biosciences), according to the manufacturer’s instructions. Pelleted EVs were resuspended in PBS and stored at −80°C. Protein content was determined by the Bradford method, using bovine serum albumin as standard.

### Scanning electron microscopy

For scanning electron microscopy (SEM) examination, EVs were fixed in 2.5% glutaraldehyde for 15 min at room temperature, washed twice with large volume of water using Vivaspin concentration devices (300,000 Da cut-off), then sedimented on glass coverslips and allowed to dry at room temperature. SEM images were obtained using a field emission gun electron scanning microscope (LEO 1525 Zeiss; Thornwood, NY, USA) after Cr metallization using a high-resolution sputter Q150T ES-Quorum apparatus (24 sec. sputter at a current of 240 mA). Chromium thickness was ~10 nm.

### Dynamic Light Scattering (DLS)

Size distribution of EVs was assessed by DLS measurements. EVs were diluted in an appropriate volume of PBS, filtered at 0.22 μm and transferred for the analyses into a dust-free cylindrical quartz cell of 10 mm inner diameter. The measuring cell was immersed in decahydronaphthalene used as an index matching liquid. Once the sample temperature was stabilized at T = 20.0 ± 0.1°C, measurements were performed using a Brookhaven Instruments apparatus (BI 9000AT logarithmic digital correlator and BI 200 SM goniometer) equipped with single mode Cobolt Samba TM solid-state laser operating at λ = 532 nm and a photomultiplier detector. The scattered light was collected at an angle of 90° and was analyzed in homodyne mode. The particle size distributions were determined using the sphere approximation for a dilute suspension, by means of the CONTIN routine, a commonly used constrained regularization method [23]. Results showed the existence of a single relaxation time, i.e. a monomodal population distribution. Data were successively processed and confirmed by the third order cumulants analysis.

### Immunoblotting

Cells were lysed at 4°C in RIPA buffer (50 mM Tris-HCl pH 8, 150 mM NaCl, 1% (v/v) NP-40, 0.1% (w/v) SDS, 0.5% (w/v) sodium deoxycholate) in the presence of a protease inhibitor mixture. Insoluble material was removed by centrifugation at 13,000 x g for 10 min at 4°C. Cell lysates or EV preparations containing an appropriate amount of proteins (5–30 μg for cell extract, 3–10 μg for EVs) were mixed with sample buffer 5X (1M Tris-HCl pH 6.8, 5% (w/v) SDS, 6% (v/v) glycerol, 0.01% (w/v) Bromophenol blue) without DTT (non-reducing conditions, used for CD9 and CD63 detection according to manufacturer’s instructions) or with 125 mM DTT (used for the rest of antibodies). In both cases samples were boiled for 5 min, electrophoresed on 12% acrylamide gel at 150 V for 1h and transferred to PVDF membrane at 100 V for 1h. As internal control, actin was used in both non-reducing and reducing conditions. Rabbit polyclonal anti-H-Ras antibody, goat polyclonal anti-Alix antibody, mouse monoclonal anti-Tsg101 antibody, mouse monoclonal anti-CD81 antibody, goat polyclonal anti-calnexin antibody, rabbit polyclonal anti-Rab5B antibody, mouse monoclonal anti-LAMP2 antibody were from Santa Cruz Biotechnology (Santa Cruz, USA), mouse monoclonal anti-CD9 and mouse monoclonal anti-CD63 were from Abcam (Cambridge, UK), mouse monoclonal anti-flotillin 1 was from BD Biosciences (Franklin Lakes, USA), mouse monoclonal anti β-actin was from Sigma-Aldrich (St Louis, USA). Sheep anti-goat (Sigma), donkey anti-rabbit and sheep anti-mouse HRP-linked secondary antibodies (GE Biosciences, Piscataway, USA) were probed according to manufacturer’s instructions. Immunoblots were detected by chemiluminescence using ECL system (GE Biosciences).

### Quantitative PCR

RNA was extracted using Trizol reagent (Sigma) according to the manufacturer’s instructions. 1 μg of RNA was reverse-transcribed into cDNA using random hexamers and SuperScript II Reverse Transcriptase (Life Technologies, Carlsbad, CA, USA). cDNA was used to determine Alix, Tsg101, CD9, CD63, CD81 transcripts by qRT-PCR in a Stratagene Mx3000P Q-PCR machine (Agilent Technologies, La Jolla, USA). Reactions were performed in triplicate using Brilliant II SYBR Green Q-PCR Master Mix (Agilent Technologies). Primers used for the amplification were 5’- GGA GGT GTT CCC TGT CTT GG (forward) and 5’- CAG CAA GGG CAC GATT GAT T (reverse) for Alix, 5’-TCA TTC CCA CAG CTC CCT TA (forward) and 5’-ACC GGC AGT CTT TCT TGC TT (reverse) for Tsg101, 5’-CTG GGA CTG TTC TTC GGC TT (forward) and 5’-GAT GGC TTT CAG CGT TTC CC (reverse) for CD9, 5’-CCT GTG CAG TGG GAC TGA TT (forward) and 5’-GAC AGA AAG ATG GCA AAC GTG A (reverse) for CD63 and 5’-GGC CGT GGT GGA TGA TGA CGC (forward) and 5’-GCA GTC CTC CTT GAA GAG GTT GCTG (reverse) for CD81. β-actin (ACTB) or GAPDH genes were amplified as endogenous control using primers 5’-AGA AAA TCT GGC ACC ACA CC (forward) and 5’-GGG GTG TTG AAG GTC TCA AA (reverse) or 5’-GAG AAG GCT GGG GCT CAT TT (forward) and 5’-AGT GAT GGC ATG GAC TGT GG (reverse) respectively. Data were analyzed using the ΔΔCt method. ΔCt was calculated subtracting the average Ct value of ACTB or GADPH to the average Ct value of Alix, Tsg101, CD9, CD63, or CD81 gene for each sample. ΔΔCt is the difference between the ΔCt for each sample and the ΔCt of mock transfected fibroblasts as control. The reported fold expression, expressed as RQ (relative quantity), was calculated by 2-ΔΔCt.

### Cells and vesicles preparation for lipidomic profile

For lipidomic analysis, cells selected with blasticidin-S as previously described were trypsinized, washed twice with PBS at 4°C and centrifuged again. Approximately 3.6 x 10^6^ of either H-RasV12 expressing cells or pcDNA6 vector transfected control cells were pelleted and stored at −80°C prior to analysis. Total cellular lipids were extracted from 9 cell pellets coming from 3 different preparations and protein concentration determined in each sample to normalize lipid content. In the case of EVs, they were obtained from cell culture medium of the 3 different preparations used for cell lipid extraction and pooled. An amount of EVs corresponding to 8 μg of proteins was used for each analysis. Lipid extraction was carried out as previously reported [24,25]. Extracts were dried under nitrogen and resuspended in methanol prior to be submitted for analyses.

### Lipid profile by liquid chromatography-tandem mass spectrometry (LC-MS/MS)

For the quantification of the different phospholipid families the MS analysis was performed with a flow injection analysis-tandem mass spectrometry (FIA-MS/MS) method. The identity of the different phospholipid families was confirmed using pure standards, namely one for each family. Methanolic extracts of either cells or vesicles were analyzed by a 3min run in both positive and negative ion mode with a 268 multiple reaction monitoring (MRM) transition in positive mode and 88 MRM transition in negative mode. An ESI source connected with an API 4000 triple quadrupole instrument (AB Sciex, USA) was used. The mobile phase was 0.1% formic acid in MeOH for FIA positive analysis and 5 mM ammonium acetate pH 7 in MeOH for FIA negative. MultiQuant^™^ software version 3.0.2 was used for data analysis and peak review of chromatograms. Quantitative evaluation of phospholipid families was performed based on standard curves. Quantitative data were normalized on the protein content of cells or vesicles.

### Statistical analysis

Statistical comparison was performed using Student’s t-test. Differences were considered statistically significant when p<0.05. For lipid analysis, 9 independent experiments for cells and 6 for EVs were carried out. To perform and graph charts of Principal Component Analysis (PCA), the XLSTAT (Addinfosft) software was used. First, the NIPALS algorithm was used to predict few missing data, then a Pearson’s correlation matrix was generated and used to calculate the significant principal components.

## Results

### Characterization of EVs released by control and H-RasV12 expressing fibroblasts

H-RasV12 was expressed in human dermal fibroblasts (HuDe) by transfection followed by pharmacological selection, using empty vector as control. H-RasV12 expression induced cell proliferation arrest and morphological changes indistinguishable from cellular senescence, indicating that tumor suppressor pathways were not ablated [17,26,27]. In fact, H-RasV12 fibroblasts displayed a typical OIS phenotype as measured by the activity of senescence associated β-galactosidase and the detection of DNA damage response (DDR) by γH2AX immunodetection. Besides, as expected in OIS, H-RasV12 overexpression was associated with higher levels of p53, p21 and p14, without evidence of H-RasV12 associated apoptosis (S1 Fig) [28–30].

**Fig 1:**
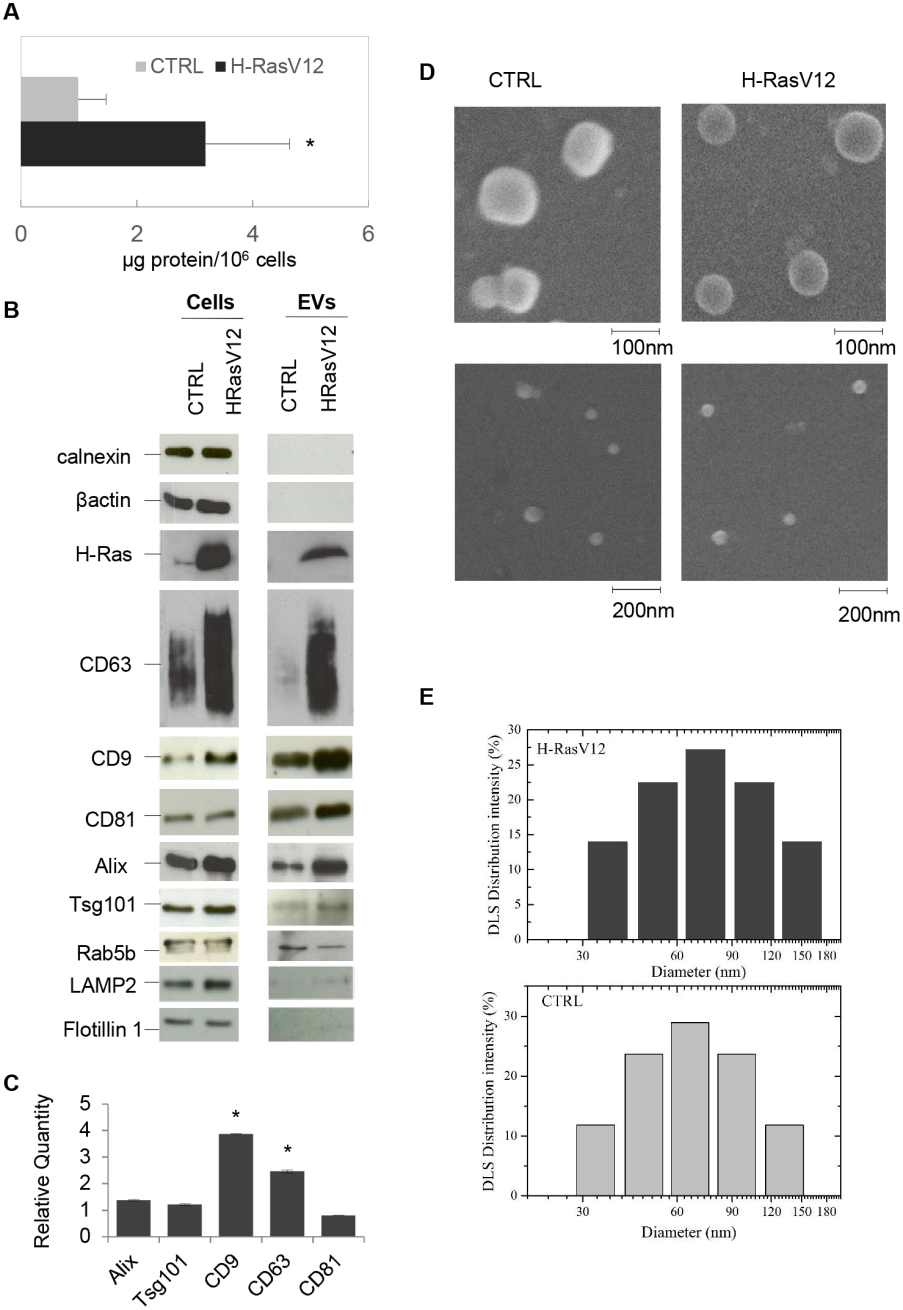
Characterization of EVs released from H-RasV12 expressing fibroblasts. Panel ***A***) EVs were isolated from H-RasV12 expressing fibroblasts and cells transfected with the vector alone as control (CTRL). Recovered EVs were quantified in μg protein/10^6^ cells. Values are the mean±S.D. of four preparations (*p<0.05). Panel ***B***) Cell extracts (30μg) and EV preparations (3μg) were separated by SDS-PAGE, electrotransferred and probed with the indicated positive and negative markers. Panel ***C***) Gene expression analysis by qRT-PCR. Ten ng of each cDNA were used as template. Reactions were performed in triplicate, using SYBR green to detect amplification. The ACTB gene was used as endogenous control. The fold expression in H-RasV12 fibroblasts with respect to CTRL is displayed. The value is expressed as Relative Quantity (RQ). The analysis was repeated three times in triplicate. The mean ± S.D. of a representative experiment is reported (*p<0.05). Panel ***D***) Scanning electron micrographs of EVs. Samples were fixed with 2.5% glutaraldehyde in PBS, sedimented onto glass coverslips and then allowed to dry at room temperature. ***E***) Histograms of the particle size distribution of EVs as determined by DLS, using the CONTIN software. Data were analysed using the cumulant fitting procedure. The following results were obtained: for H-RasV12, a hydrodynamic diameter D_h_ = (85±16)nm and a distribution width σ_h_ = (73±5)nm (n = 3), adjusted R^2^ > 0.81; for control cells, a hydrodynamic diameter D_h_ = (80±13)nm and a distribution width σ_h_ = (60±3) nm (n = 3), adjusted R^2^ > 0.93.

EVs released in the cell culture medium were isolated from fibroblasts transfected either with H-RasV12 or pcDNA6 vector alone as a control, using the polymer co-precipitation method (Exoquick-TC was used as reagent) [31]. The amount of released EVs in each preparation was assessed by measuring the total protein content (Fig 1A) and then further characterized by Nanoparticle Tracking Analysis (NTA) (S2 Fig). Results showed that H-RasV12 cells enabled the recovery of a higher amount of EVs, suggesting a higher release of EVs per cell in association with H-RasV12 expression. As EVs are usually enriched in specific proteins, we performed immunoblotting using as positive markers CD9, CD63 and CD81 tetraspanins (membrane proteins enriched in EVs), Alix and Tsg101 (ESCRT machinery proteins involved in vesicle biogenesis), Rab5b (early endosomal marker), LAMP2 (lysosomal membrane marker) and flotillin 1 (plasma membrane microdomain marker), in agreement with guidelines [32]. We used calnexin, an endoplasmic reticulum (ER) protein usually not present in EVs, as a negative marker. Results showed that EVs contained the 3 tetraspanins, Alix, Tsg101, and Rab5b, whereas LAMP2 and flotillin 1 were barely detectable and calnexin and actin were not revealed (Fig 1B). On the other hand, the EVs obtained by ultracentrifugation, while confirming a similar enrichment of CD9, CD81, Tsg101 and Alix, presented also certain levels of calnexin and actin (S3 Fig), indicating the unwanted presence of ER components.

EVs from H-RasV12 fibroblasts clearly showed the presence of H-Ras, which was not detectable in EVs from control cells, suggesting that the mutant H-Ras protein could be transferred via EVs. Moreover, we detected higher signals for all the positive markers but Rab5b in EVs from H-RasV12 fibroblasts (Fig 1B). Higher expression of CD63, CD9, Alix and Tsg101 was also recorded in parental cells, indicating that H-RasV12 fibroblasts expressed higher levels of these proteins, which therefore resulted increased in their released EVs. As expected, proteins used for cell extracts normalization, such as calnexin and actin, were not affected by H-RasV12 expression (Fig 1B). In this regard, qRT-PCR (Fig 1C) confirmed that CD63 and CD9 transcripts, but not Alix and Tsg101, were up-regulated in H-RasV12 fibroblasts, suggesting a specific up-regulation of these tetraspanin transcripts during H-RasV12 induced senescence. The EVs released by H-RasV12 and control fibroblasts were further characterized by Scanning Electron Microscopy (SEM) analysis. In both cases, EVs displayed a characteristic cup-shape morphology usually associated with vesicles of endosomal origin, with diameters ranging from 40 to 120 nm (Fig 1D). The Dynamic Light Scattering (DLS) analysis (Fig 1E) and NTA (S2 Fig) confirmed SEM observations, showing an EV size distribution between 30 and 150 nm. Altogether, these results indicated that the obtained EV population was characterized mainly by small EVs, consistent with exosomes and/or small membrane microvesicles [4].

### Glycerophospholipid (GPL) and sphingolipid (SL) profiles of control and H-RasV12 fibroblasts, and their released EVs

The lipidomic profiles of H-RasV12 and control fibroblasts were compared to the lipidome of released EVs. The number of detected lipid species in both cells and EV samples is reported in S1 Table. The same lipid species, 228 GPL and 49 SL, were found in both cell samples. In turn, 213 and 214 GPL molecular species were detected in H-RasV12 and control EVs, respectively. The only difference was the absence in H-RasV12 EVs of phosphatidylethanolamine (PE) aa 32:5 (PEaa 32:5, a PE carrying different fatty acids bound to the glycerol moiety by two ester linkages at both *sn-1* and *sn-2* position: di-acyl form, therefore aa means acyl-acyl). The analysis of SL revealed the presence of 33 molecular species in both EV preparations. The only GPL subclass that was detected in cells but not in EVs was lysophosphatidylinositol (LPI). In addition, GM1, GM2 and GM3 gangliosides were also detected in cell samples but not in EVs. Other differences regarded the molecular species of phosphatidylinositol (PI) (13 in cells, 3 in EVs), PEaa (67 in cells, 63 in EVs), ceramide (Cer, 6 in cells, 2 in EVs).

**Fig 2:**
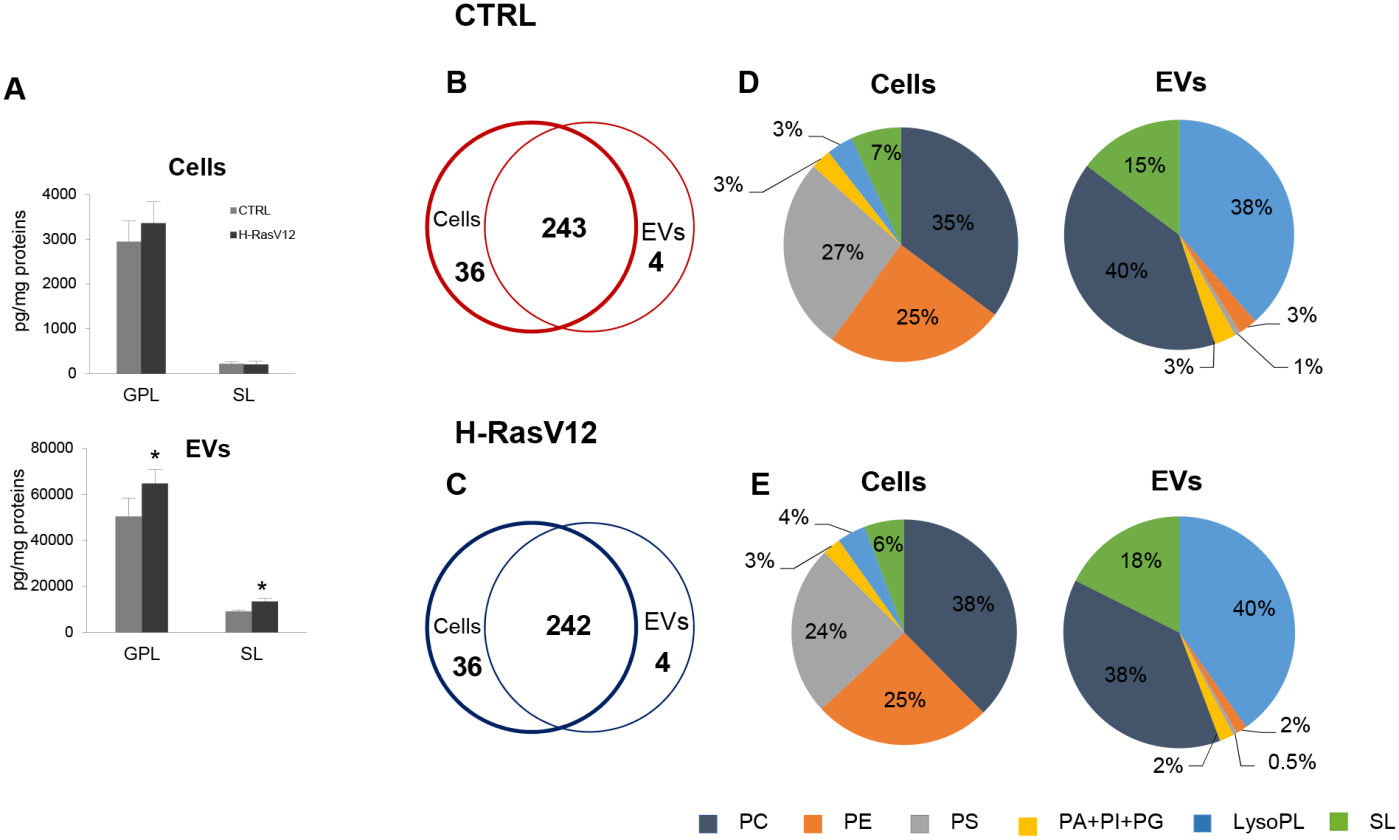
Lipid subclass composition of H-RasV12 vs control fibroblasts and H-RasV12 vs control EVs. ***A***) Amounts of GPL and SL relative to protein content. Data are reported as mean ± S.D. (n = 9, cells; n = 6, EVs) (*p<0.05, CTRL *vs* H-RasV12). ***B*** and ***C****)* Venn diagram showing the number of species detected in cells and their released EVs. ***D*** and ***E***) The composition of lipid classes of cells and their released EVs.

Further comparison between cells and EVs showed that 243 species were common, 36 species were detected only in cells and 4 species were exclusively found in EVs (Fig 2), i.e. LPC 20:3, LPC 26:1, phosphatidylcholine (PC) ae 42:4, PEae 30:0 (PC or PE with a fatty acid at the *sn-1* position linked by a vinyl ether linkage while the fatty acid at the *sn-2* position linked by an ester linkage to the glycerol moiety: alkyl-acyl form, therefore ae means acyl-alkyl; these molecules are also known as plasmalogens).

The quantitative analysis of total detected lipids highlighted significant differences between fibroblasts and EVs (Fig 2). Firstly, we observed that EVs had a higher lipid/protein ratio with respect to cells (Fig 2A). Interestingly, EVs from H-RasV12 fibroblasts had a higher level of GPL and SL per μg of protein, as compared to those released from control cells (≈15%). Second, GPL and SL distributions were different between cells and EVs. In cells, GPL represented ≈93% of the total detected lipids, while SL accounted for ≈7%. In the case of EVs, ≈84% and ≈15% of the total detected lipids were GPL and SL, respectively. This increase of protein-normalized lipid content and the higher percentage of SL in EVs is consistent with previous lipidomic studies [14,15].

**Fig 3:**
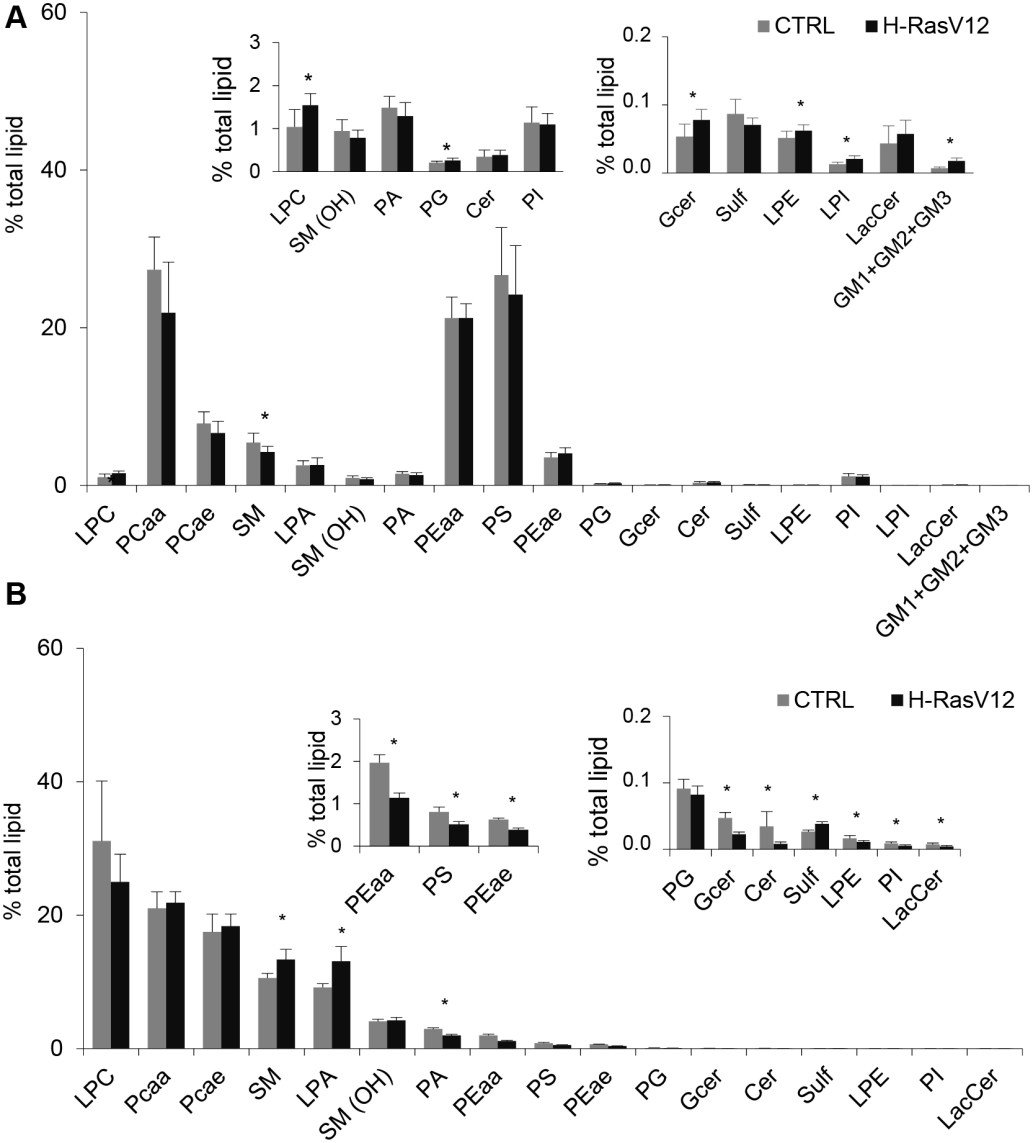
Lipid classes percentage in control and H-RasV12 cells (*A*) and their released EVs (*B*). Lipid extracts from control and H-RasV12 fibroblasts and their released EVs were analysed by LC/MS-MS. The amount of each lipid class is expressed as percentage of the sum of all identified lipids. Data are reported as mean ± S.D. (n = 9, cells; n = 6, EVs) (*p<0.05, CTRL *vs* H-RasV12). The inserted panels expand the vertical axis to allow the comparison of low abundance lipid subclasses.

The comparative analysis of cells vs EVs lipid subclasses showed that EVs have a peculiar composition as compared to the cells of origin, independently of H-RasV12 expression. The main differences between cells and EVs were: i) the amount of lyso-PL in EVs, which was 10 times more abundant in EVs with respect to parental cells; ii) the high percentage of PS (≈27%) and PE (≈25%) in cells, as PS and PE were both detected in EVs, but the amount was much lower (≈3% for PS and ≈2% for PE) (Fig 2). Further comparative analysis showed that, despite the similar percentage of total PC (PCaa+PCae) in EVs and cells of origin, EVs contained a higher percentage of PC-derived plasmalogens (≈18%) in comparison with parental fibroblasts (≈8%) (Fig 3A and 3B). The presence of SM(OH) was also clearly higher in EVs (≈4% of total detected lipids) than in cells (less than 1%) (Fig 3A and 3B).

Moreover, we compared the lipid subclass composition of H-RasV12 vs control fibroblasts to specifically detect changes associated with H-RasV12 induced senescence (Fig 3). In both cell samples, the most abundant lipid classes were PC (≈35%), followed by PS (≈27%), PE (≈25%) and SM (≈5%) (Fig 3A). However, specific differences between H-Ras-V12 and control fibroblasts were observed, as the SM level was significantly decreased in H-RasV12 fibroblasts, whereas lyso-PL (LPE, LPC, LPI), phosphatidylglycerol (PG), glucosyl/galactosyl-ceramide (GCer), GM2 and GM3 levels were higher (Fig 3A).

When the composition of EVs released from H-RasV12 and control fibroblasts was compared, we observed that in both samples the most represented lipid subclass was LPC (≈30%), followed by PCaa (≈20%), PC-derived plasmalogens (≈18%), SM (≈10%) and lysophosphatidic acid (LPA) (≈9%). However, specific differences between control and H-Ras-V12 EVs were observed: SM, LPA and sulfatides (Sulf) were increased in EVs released from H-RasV12 cells, whereas phosphatidic acid (PA), PE, PS, PI, LPE, Cer, GCer and lactosyl-ceramide (LacCer) were reduced (Fig 3B).

### Analysis of GPL molecular species of H-RasV12 vs control fibroblasts, and their released EVs

**Fig 4:**
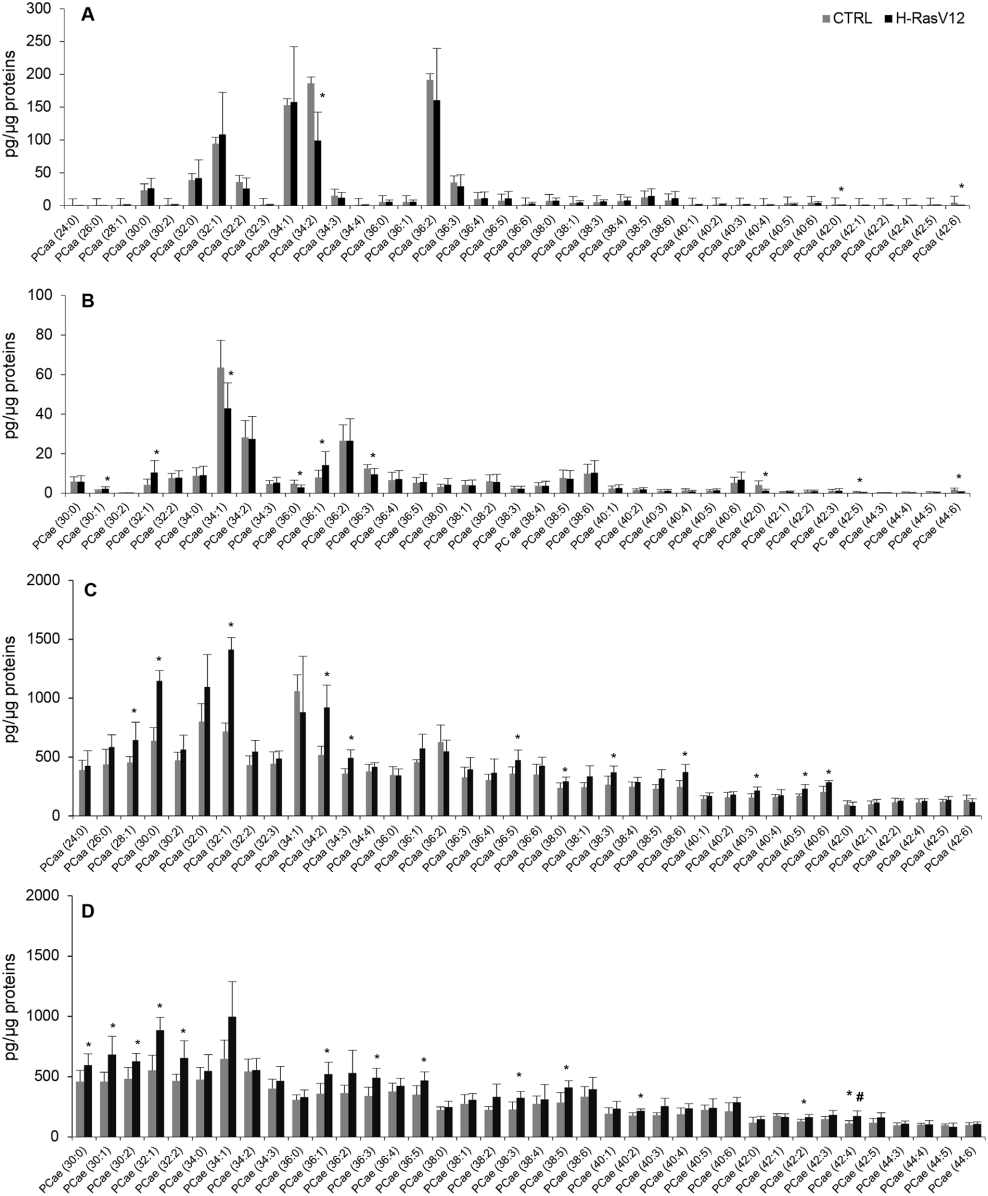
Molecular species of PCaa and PCae in H-RasV12 and control fibroblasts, and their released EVs. Lipid extracts from control and H-RasV12 cells (***A*, *B***) and their released EVs (***C*, *D***) were analysed by LC/MS-MS. Data are expressed as pg of lipid species/μg of proteins. Mean values ±S.D. (n = 9, cells; n = 6, EVs) are shown (*p<0.05, CTRL *vs* H-RasV12; # lipid species exclusively found in EVs).

To unveil differences between H-RasV12 and control fibroblasts, and between H-RasV12 and control EVs, we focused the attention on the relative abundance of individual molecular species within each subclass. In cells, a few significant differences in PCae and PCaa content between control and H-RasV12 samples were found, i.e. the decrease of 3 PCaa molecular species in H-RasV12 fibroblasts (Fig 4A), and changes in 10 molecular species of PCae (3 increased and 7 decreased in H-RasV12 fibroblasts) (Fig 4B). These findings indicated that H-RasV12 expression was associated with a few rearrangements of acyl and alkyl chains in HuDe cells. In EVs, 12 out of 38 PCaa molecular species and 13 out of 38 PCae molecular species showed significant differences (Fig 4C and 4D), as they were all increased in the vesicles released from H-RasV12 cells. The comparison of cells vs EVs also showed a wider distribution of different molecular species of PCaa and PCae in EVs as compared to cells, in which most of the PCae and PCaa content was due to a few lipid species.

**Fig 5:**
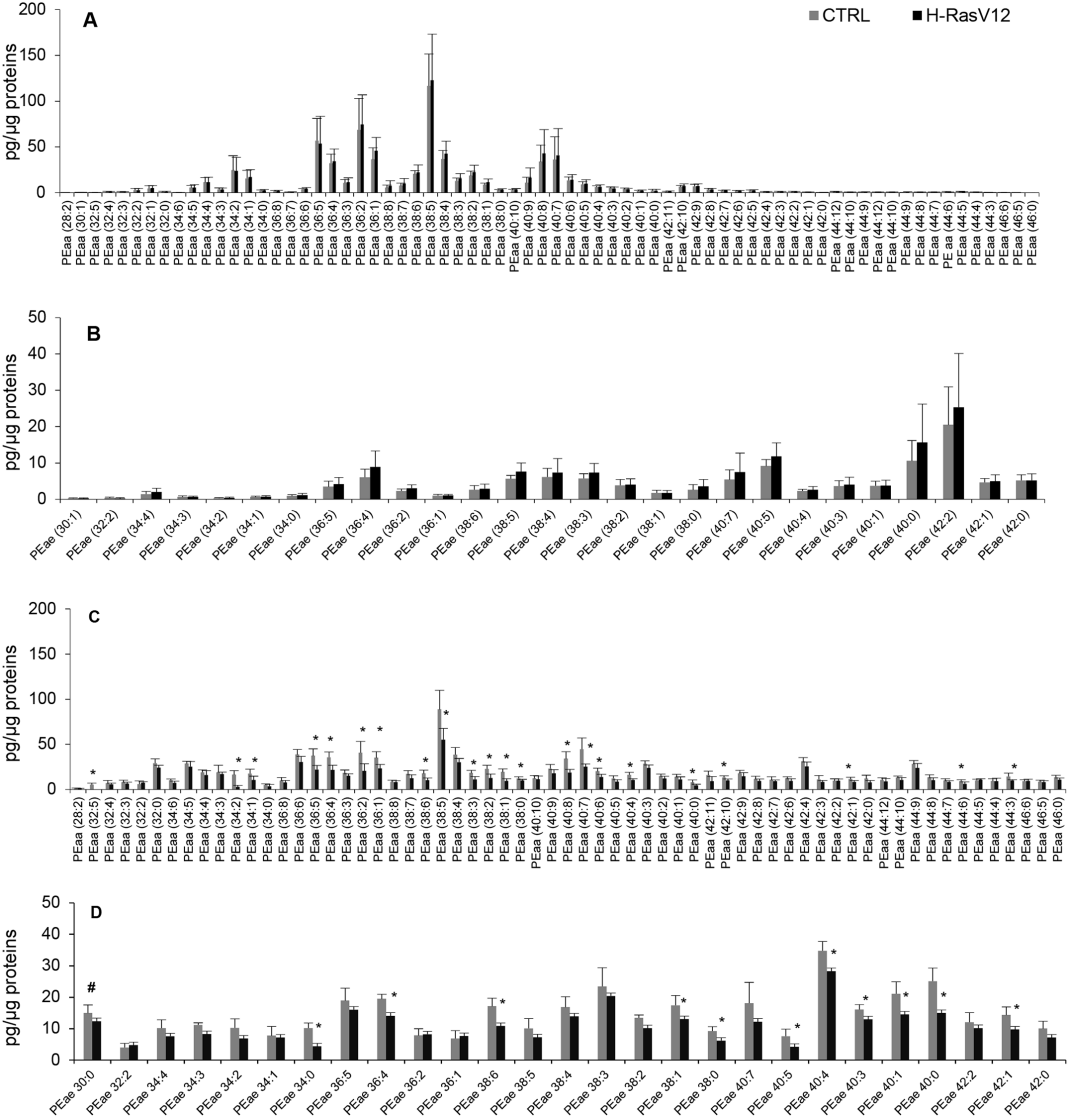
Molecular species of PEaa and PEae in H-RasV12 and control fibroblasts and their released EVs. Lipid extracts from H-RasV12 and control fibroblasts (***A*, *B***) and their released EVs (***C*, *D***) were analysed by LC/MS-MS. Data are expressed as pg of lipid species/μg of proteins. Mean values ±S.D. (n = 9, cells; n = 6, EVs) are shown (*p<0.05, CTRL *vs* H-RasV12; # lipid species exclusively found in EVs).

As previously reported, the content of PE was lower in EVs with respect to cells. Detailed analysis revealed no significant quantitative differences in any individual molecular species between H-RasV12 and control fibroblasts, despite the remarkable detection of 67 PEaa and 28 PEae (Fig 5A and 5B). In EVs, we noticed that 22 out of 63 PEaa species were decreased in H-RasV12 EVs, together with 11 out of 27 PEae species (Fig 5C and 5D).

In cells, the analysis of the PS, PI, PG and PA detected species (S4 Fig) revealed no relevant quantitative differences between H-RasV12 and control samples. In EVs, 3 out of 9 PS species and 3 out of 9 PA species were significantly decreased in H-RasV12 samples, whereas regarding PG, only 32:1 was increased and no change was observed for PI.

### Analysis of lyso-PL subclass molecular species in H-RasV12 vs control fibroblasts, and their released EVs

**Fig 6:**
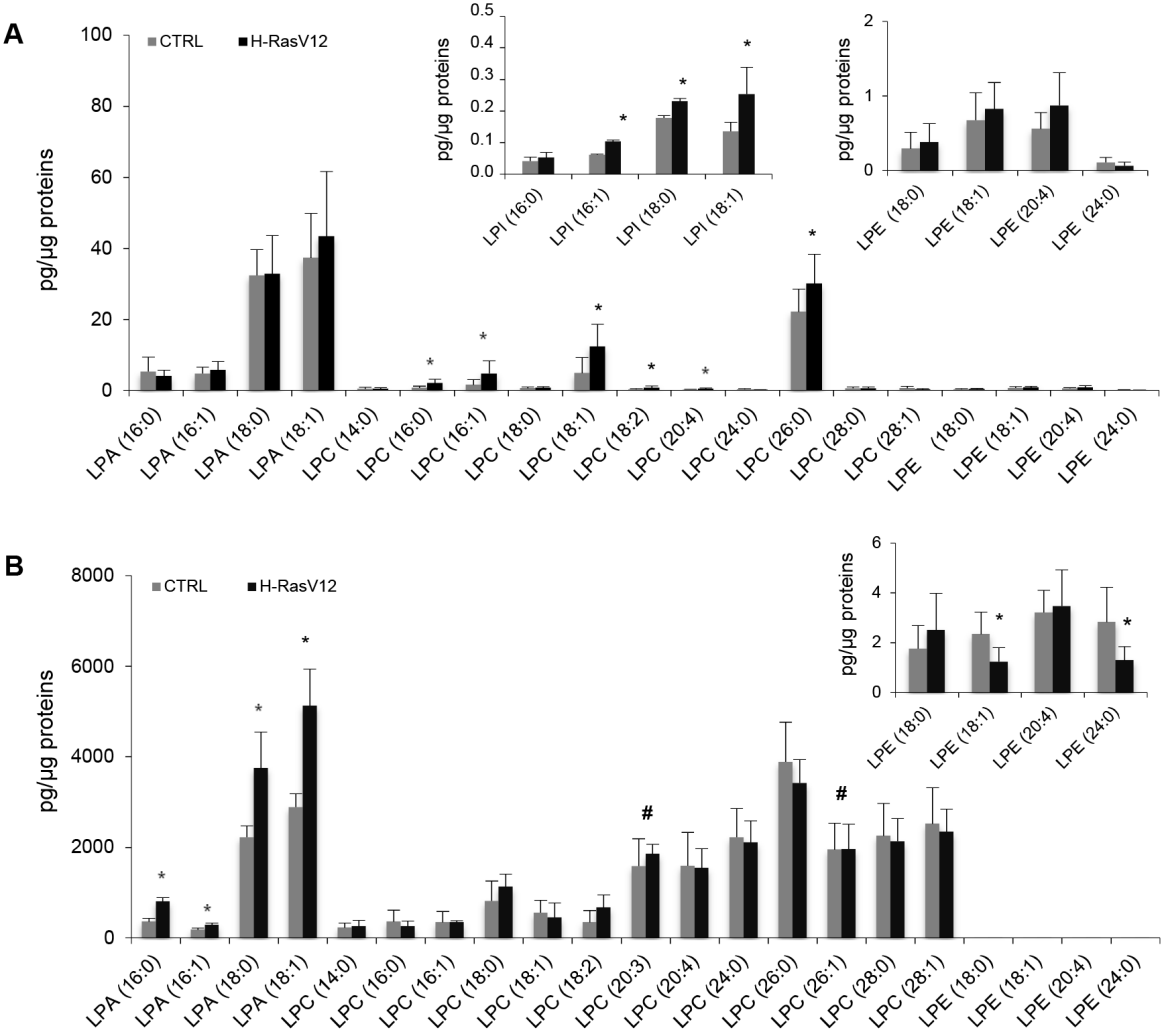
Molecular species of lyso-PL subclasses of H-RasV12 and control fibroblasts (*A*), and their released EVs (*B*). Lipid extracts from H-RasV12 and control cells and their released EVs were analysed by LC/MS-MS. Data are expressed as pg of lipid species/μg of proteins. Mean values ±S.D. (n = 9, cells; n = 6, EVs) are shown (*p<0.05, CTRL vs H-RasV12; # lipid species exclusively found in EVs). The inserted panels expand the vertical axis to allow comparison of low abundance lipid subclasses.

In H-RasV12 expressing fibroblasts, analysis of LPC showed that 6 out of 11 molecular species were significantly increased (16:0; 16:1; 18:1; 18:2; 20:4 and 26:0) with respect to control cells (Fig 6A). In EVs, no individual molecular species of LPC was significantly different between H-RasV12 and control EVs (Fig 6B). The comparison between the individual species detected in cell and EV samples showed that in fibroblasts the most abundant LPC species was LPC 26:0, while all the other species were present at a very low amount (Fig 6A). In EV samples, LPC 26:0 was still abundant, but LPC with acyl chains ≥20 carbons were present at a comparable level (Fig 6B).

The level of LPA and LPE molecular species remained unchanged in H-RasV12 fibroblasts with respect to control cells (Fig 6A), whereas 3 out of 4 LPI molecular species were present at higher level (Fig 6A). In H-RasV12 EVs, the 4 species of LPA were all significantly increased, whereas 2 out of the 4 LPE species were reduced (Fig 6B).

### Analysis of SL subclasses molecular species in H-RasV12 vs control fibroblasts, and their released EVs

**Fig 7:**
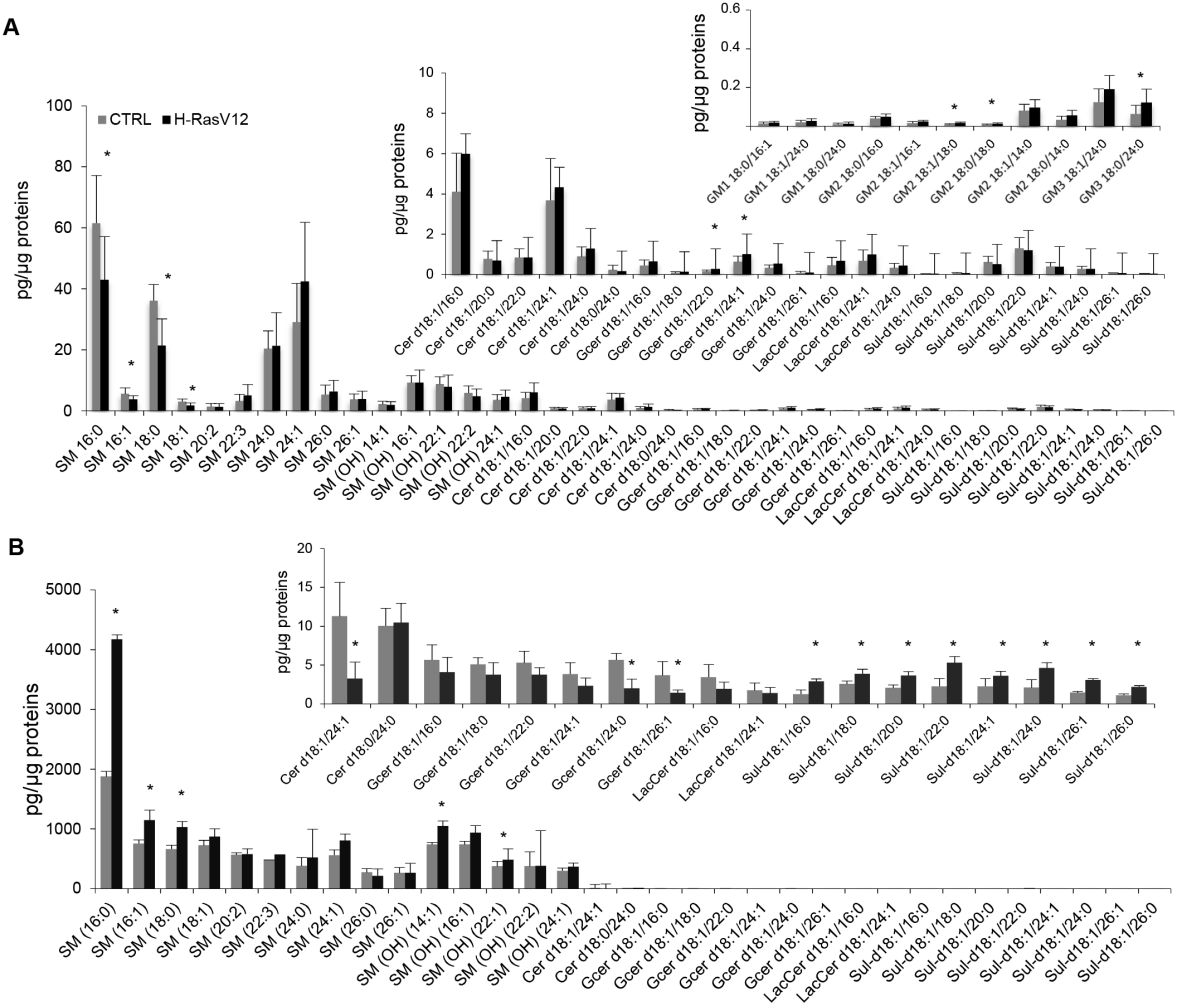
Molecular species of sphingolipid subclasses of H-RasV12 and control fibroblasts (*A*), and their released EVs (*B*). Lipid extracts from H-RasV12 and control cells and their released EVs were analysed by LC/MS-MS. Data are expressed as pg of lipid species/μg of proteins. Mean values ± S.D. (n = 9, cells; n = 6, EVs) are shown (*p<0.05, CTRL vs H-RasV12). The inserted panels expand the vertical axis to allow comparison of low abundance lipid subclasses.

In H-RasV12 cells, quantitative differences in several SL subclasses with respect to control cells were observed (Fig 7A). In particular, a reduced content of SM in H-RasV12 fibroblasts was measured, which was due to a decreased content of 4 molecular species (16:0; 16:1; 18:0; 18:1) (Fig 7A). In EVs, the most abundant molecular species was SM 16:0, which accounted for 1876±143pg/μg of proteins in control EVs and doubled up to 4171±475pg/μg of proteins in H-RasV12 EVs (Fig 7B). Other species increased in H-RasV12 EVs were SM 16:1 and SM 18:0 (Fig 7B). Remarkably, in cells and EVs we also detected hydroxylated SM (Fig 7) and two SM(OH) molecular species, 14:1 and 22:1, were significantly increased in EVs from senescent fibroblasts (Fig 7B).

The analysis of other SL subclasses revealed that the level of GCer and GM2/GM3 were increased in H-RasV12 fibroblasts (Fig 3). This was mostly due to the significant increase of 2 molecular species of GCer, 2 of GM2 and 1 of GM3 (Fig 7A). In EVs, gangliosides could not be detected (Fig 7). Besides, 1 out of 2 Cer and 2 out of 6 GCer species were decreased in vesicles from H-RasV12 fibroblasts (Fig 7B). Noteworthy, the 8 sulfatides species detected did not change between H-RasV12 and control fibroblasts, but all significantly increased in H-RasV12 EVs, indicating a rearrangement of sphingolipids in vesicles, with a decrease in Cer and GCer in favour of an increase in Sulf (Fig 7B).

### PCA of cellular and vesicular samples

**Fig 8:**
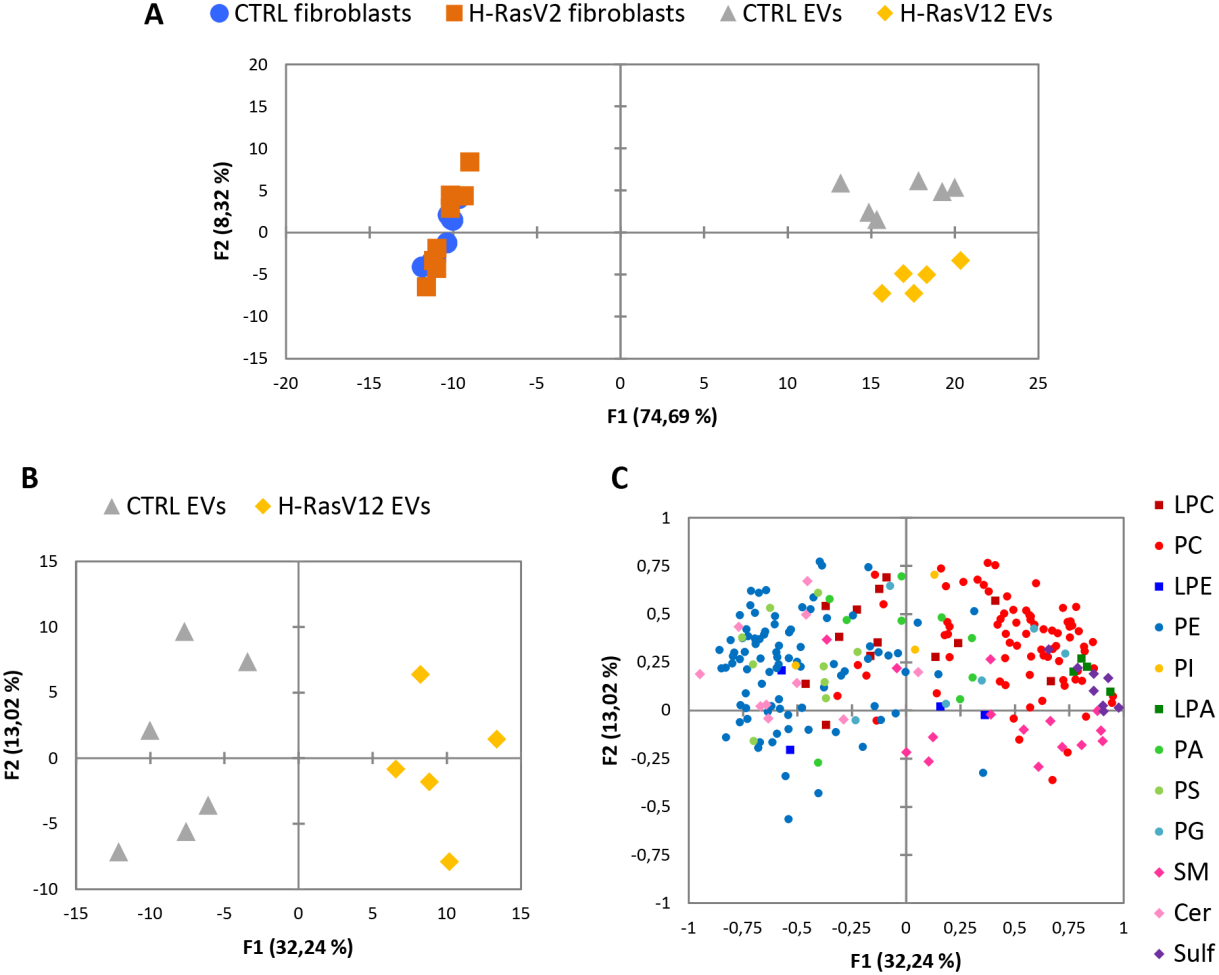
Multivariate analysis by PCA. ***A***) PCA score plot for lipid species data from all the study samples revealed different clustering between cells and EVs. ***B***) Score plot generated by PCA analysis of EVs released from H-RasV12 and control fibroblasts shows the two clusters. ***C***) Loading plot of EVs released from H-RasV12 and control fibroblasts shows the relevance of lipid species for the clustering.

PCA analysis was performed in order to cluster the obtained data for an accurate analysis of the results. Fig 8 illustrates the score plot, which reports the first principal component (F1) versus the second one (F2), together accounting for about 83% of the total sample variance. Cells and EVs cluster in two experimental groups based on the first principal component (F1), and the second component (F2) allows the separation between H-RasV12 and control EVs. Therefore, despite the differences in the level of individual molecular species observed between H-RasV12 and control fibroblasts, the global analysis of lipid content could not discriminate these two samples, even if it could separate EVs from their parental cells. The PCA analysis of EVs clearly indicated that the global lipid analysis was more successful in discriminating EVs released by H-RasV12 fibroblasts from those released by control cells (Fig 8B). In addition, the variables mainly responsible for the formation of H-RasV12 and control clusters were Sulf and a few species of SM, PC and PE (Fig 8C).

## Discussion

The release of EVs may modulate lipid mediated signaling in the extracellular environment, being a key factor in the determination of the amount and type of lipids available to neighbor cells. To shed light on how H-RasV12 expression in human fibroblasts influences cell lipid composition, affecting the lipid content of EVs, we investigated the lipid profile of H-RasV12 and control fibroblasts compared to that of the respectively released EVs.

The biophysical characterization of EVs from H-RasV12 and control samples showed that they shared a similar morphology and size distribution. Besides, H-RasV12-induced senescence was associated with an increased expression of CD63 and CD9, two tetraspanins highly enriched in EVs and considered the best EV markers currently available [33,34]. In agreement with previous studies on EVs lipid profile [14,15], the analysis of the lipid content showed a peculiar sorting of cellular lipids into EVs. Firstly, EVs displayed a higher lipid/protein ratio with respect to cells. Furthermore, the lipid composition of EVs compared to that of cells revealed that EVs contain a higher level of sphingolipids with respect to glycerophospholipids. Besides, EVs showed a high content of lyso-PL, which was 10 times larger in EVs with respect to their parental cells, consistently with previous studies [13, 15].

Most of the lyso-PL in EVs consisted of LPC and LPA. Interestingly, these species are not only metabolites in membrane phospholipid synthesis and degradation, but also ubiquitous bioactive molecules influencing a broad variety of biological processes by binding to cognate G protein-coupled receptors (GPCRs) [35]. Noteworthy, it has been recently reported that the treatment of cells with LPC increases EV release [36]. Therefore, the presence of LPC and LPA in vesicles released outside the cells not only suggests the possible activation of lyso-PL receptors mediated signalling in target cells, but also indicates a likely mechanism of signal amplification. In this context, a very recent study has also shown that autotaxin, the secreted lysophospholipase hydrolysing LPC to produce LPA, binds to the surface of EVs outside the cell. In this way, vesicular LPC can be converted into LPA, which can be locally released to activate the LPA receptors on the surface of target cells [37]. We detected the presence of autotaxin in both control and H-RasV12 EVs, with a certain prevalence in H-RasV12 EVs (S5 Fig). The presence of lyso-PL in EVs has been previously observed [10,14,15,38,39]. However, in EVs released by colorectal cancer cells, LPE and LPS were more abundant than LPC [15], whereas LPC was the most abundant lyso-PL subclass in EVs released by platelets, together with LPA [38,39]. These evidences suggest that the enrichment in specific lyso-PL subclasses could be dependent on the type and physiological/pathological status of the cell.

EVs lipid composition also showed an enrichment in ether-linked PC with respect to parental cells (about 2-fold). The presence of ether-linked PL in EVs has been previously reported by a few studies [15,39]. These lipids are implicated in the regulation of many physiological events, for example they may serve as a depot of lipid mediators [40] and are involved in membrane fusion and trafficking [41]. Furthermore, Phuyal et al. [42] have recently reported that the ether lipid precursor hexadecylglycerol stimulates the release and changes the composition of EVs derived from prostate cancer cells. Therefore, once again EVs appear enriched with lipids that could further stimulate the release of EVs from target cells.

The higher presence of hydroxylated SM species was another peculiarity of EVs with respect to parental cells. Sphingolipids often contain hydroxylated acyl chains and 2-hydroxylation is known to affect interlipid association, leading to the stabilization of membrane, as in the case of myelin for the nervous system [43]. However, information about the physiological role of hydroxylated SM outside the nervous system is relatively poor, so it is difficult to speculate on their function in EVs.

The search for lipid species highly enriched or exclusively present in EVs is an attractive field, as these molecular fingerprints could be useful as markers of EVs isolated from different body fluids, able to discriminate them from the tissue of origin. In depth analysis of single lipids revealed that 4 species (LPC 20:3; LPC 26:1; PCae 42:4; PEae 30:0) were detected only in EVs. However, the metabolic route of their synthesis and thus the biological meaning of their presence in EVs must be further assessed.

H-RasV12-induced alteration in the lipid profile of fibroblasts and their released EVs could allow the identification of specific oncogene-induced senescence signatures. In H-RasV12 fibroblasts, taking into account the highly abundant lipid species (i.e. more than 1% of total detected SL and GPL), we observed a significant decrease of SM percentage, mainly due to the lower level of the four molecular species of SM with shorter acyl chains, and an increase of LPC. SM is synthesized by the enzymatic transfer of phosphocholine from phosphatydilcholine to ceramide by sphingomyelin synthase. In turn, ceramide is de-novo synthesized by condensation of palmitate and serine, catalysed by serine palmitoyl transferase. Conversely, SM is degraded into ceramide by several sphingomyelinases (SMases), which include acid SMase and neutral SMase. We did not observe any significant difference in the content of ceramide that could reflect changes in SM de-novo biosynthesis or degradation. However, SMases have been previously involved in EV biosynthesis. In fact, the pharmacological inhibition of nSMase reduces the release of exosomes [44], whereas rapid activation of aSMase triggers vesicle release from glial cells [45]. On this basis, we determined nSMase and aSMase activity in H-RasV12 and control fibroblasts, but we did not observe any significant difference (S6 Fig). Therefore, further comprehensive investigation is required to explain the change in SM content. In the case of LPC, we observed an increase of lyso-PL in H-RasV12 fibroblasts, mainly due to higher level of LPC but also of LPE and LPI. This increase could be related to an enhanced activity of phospholipase A2/A1, that has been previously reported on Ras-transformed cells [46]. Interestingly, a recent investigation on senescing human bone marrow-derived mesenchymal stem cells specifically identified a few LPC and LPE species as senescence markers [47], suggesting their use as indicators of senescence in cultured stem cells. Complex glycosphingolipids which are present in small amount (less than 0.1%), i.e. GCer, GM2 and GM3, were increased in H-RasV12 fibroblasts. As these sphingolipids are signal molecules involved not only in senescence but also in apoptosis and autophagy, this rearrangement seems to be linked to the regulation of specific signalling pathways upon H-RasV12 activation.

The comparison of EVs released by H-RasV12 and control cells show many differences associated with H-RasV12-induced senescence. The structural lipid core, represented by choline-containing glycerophospholipids, was similar in both EV samples. However, all the other lipid subclasses but SM(OH) and PG, differed between H-RasV12 and control EVs. Among the most represented lipid species (i.e. more than 1% of total EV detected lipids), SM and LPA were increased, whereas PA, PE and PS were decreased. The correlation between the increase in LPA and the decrease in PA in H-RasV12 EVs with respect to control EVs suggested an activation of phospholipase A2 (or phospholipase A1) enzymatic activity towards PA in EVs. As a matter of fact, the presence in EVs of 3 phospholipase A2 classes (the calcium-dependent cPLA2-IVA, the calcium-independent iPLA2-VI A, and the secreted sPLA2-II A and V) has been previously reported [16]. However, in addition to being generated by phospholipase A2/A1 activity on PA, LPA could be also generated by the action of the above mentioned lysophospholipase D autotaxin, which removes the choline groups from LPC (S5 Fig).

As reported above, the increase of SM in EV could be due to a higher production by SM synthases or a lower degradation by SMases. However, an EV-associated activity of enzymes involved in sphingolipid metabolism has never been reported. In previous studies showing the involvement of a ceramide-dependent mechanism in the release of EVs upon specific treatments, these EVs were also shown to be enriched in ceramide [48,49]. Nevertheless, in our study, a ceramide enrichment in EVs vs cells was not detected (less than 0.1% in EVs and about 1% in cells), and the ceramide content was decreased in H-RasV12 vs control EVs. On the other hand, H-RasV12 EVs displayed a higher content of proteins involved in ESCRT-dependent biogenesis (Alix, Tsg101) and a remarkable higher content of tetraspanins (CD9 and CD63), which suggested a potentiation of ESCRT-dependent and tetraspanin-dependent EV secretion routes. As tetraspanins are usually associated to sphingolipid and cholesterol enriched domains, their higher presence might contribute to the higher level of SM detected in EVs from H-RasV12 fibroblasts [50].

Less represented lipid species (i.e. accounting for less than 0.2% of total detected lipids) were all decreased in EVs from H-RasV12 fibroblasts, except for sulfatides. These are synthesized from ceramide by two transferases (ceramide galactosyltransferase and cerebroside sulfotransferase) and degraded into lysosomes by arylsulfatase A. Due to the lower level of Cer and Gcer in H-RasV12 EVs, neither an increased conversion by ceramide galactosyltransferase and cerebroside sulfotransferase or a lower degradation by arylsulfatase A could be excluded. The increase in sulfatides is of interest, as even if these sphingolipids are present at a very low level, their net negative charge could certainly affect the overall charge of EVs released by H-RasV12 fibroblasts.

Processing the quantitative differences by PCA analysis allowed clustering cells and EVs in two experimental groups, indicating that the whole lipid composition can be easily used to distinguish between cellular and vesicular samples. However, this analysis failed in clustering H-RasV12 and control fibroblasts, but succeeded in separating their released EVs. Therefore, PCA results showed that the biochemical investigation of EV lipid composition, which represents a kind of extracellular compartment, can reveal more original information than the global lipid analysis of the cell, confirming the potential of EVs as source of senescence signatures and signals. Of note, investigations using a membrane fraction instead of cell total lipids might give different results and represent an important future direction of study, which might help to elucidate the lipid profile changes associated with OIS in specific subcellular compartments.

In summary, the present study highlighted that EVs show an enrichment in hydroxylated sphingomyelin, lyso- and ether-linked phospholipids compared to cells and revealed specific H-Ras-induced senescence signatures in EVs, namely sphingomyelin, lysophosphatidic acid and sulfatides.

## Supporting information

S1 Fig H-RasV12 induced senescence. A) Senescence-associated β-galactosidase staining. Microscopy images of HuDe (untransfected), CTRL (empty vector transfected) and H-RasV12 fibroblasts and quantification of SA-βgal positive cells. SA-β-gal positive cells were counted at least on three different fields in three independent experiments (*p<0.05, CTRL vs H-RasV12). B) Immunoblotting of OIS markers. Cell extracts (30 μg) were separated by SDS-PAGE, electrotransferred and probed with mouse monoclonal anti-p53, rabbit polyclonal anti-γH2AX, rabbit polyclonal anti-p14 ARF (Santa Cruz Biotechnology) and rabbit monoclonal anti-p21 (Cell Signaling Technology). C) Immunostaining for γH2AX. Cells were fixed in 4% paraformaldehyde, permeabilized with 0.1% Triton X-100 in PBS, incubated with an anti-γH2AX in 2% FBS/0.01% Triton X-100/PBS and labelled with an anti-rabbit Alexa-Fluor 594 antibody. Nuclei were stained with 1 μg/ml DAPI. Fluorescence microscopy analysis was carried out using a Nikon TE2000 microscope through a 60x oil immersion objective. D) Analysis of late apoptosis by detection of DNA content. Low molecular weight DNA produced by apoptosis-induced DNA fragmentation in CTRL and H-RasV12 fibroblasts was solubilized by cell permeabilization with 70% ethanol and high molecular weight DNA retained in fibroblasts quantified by DAPI staining. Cellular fluorescence was analysed by a NucleoCounter NC-3000 automated image analysis system (Chemometec) and the percentage of sub-G1 cells on total cells was quantified by NucleoView software.
(PDF)

S2 Fig Nanoparticle Tracking Analysis (NTA) of EVs released from H-RasV12 and control fibroblasts. A) Quantification of EVs. The amount of released EVs was measured by NTA and expressed as EVs/cell. Briefly, EVs pellets were resuspended in the amount of PBS (filtered through a 0.02 μm filter) needed to obtain a concentration within the recommended range (2 x 10^8^–1 x 10^9^ particles per ml) and vortexed for 1 min. Samples were then loaded into a NS500 instrument (Malvern, UK). Five videos, each of 60 s, were acquired for every sample and analysed by NTA 2.3 software. B) Determination of EVs protein content. The protein amount of EV preparations was quantified by Bradford assay and normalized for the amount of released EVs as measured by NTA, then expressed as μg protein/EV. The y-axis values are multiplied by 10^9^. C) Representative particle size distribution of EVs from H-RasV12 fibroblasts (right panel) and control cells (left panel), reported as relative particle number, i.e. number of particles of the indicated diameter with respect to the total number of analyzed particles. For H-RasV12 we found a mean diameter of 104.0 ± 3.7 nm and mode of 76.5 ± 1.8 nm; for CTRL, we found a mean diameter of 95.1 ± 2.6 nm and mode of 77.9 ± 3.7 nm.
(PDF)

S3 Fig Immunoblotting analysis of EVs prepared by ultracentrifugation. EVs were isolated from H-RasV12 expressing fibroblasts and cells transfected with the vector alone as control (CTRL) by differential ultracentrifugation. Collected medium underwent centrifugation at 2000 x g for 10 min, then at 10,000 x g for 30 min and, finally, at 100,000 x g for 70 min. The final pellet was resuspended in PBS, centrifuged again at 100,000 x g for 70 min to wash it, then the total amount of pelleted proteins was determined by Bradford method. EV preparations (3μg) were separated by SDS-PAGE, electrotransferred and probed with the positive and negative markers indicated.
(PDF)

S4 Fig Molecular species of PS, PI, PG and PA of H-RasV12 and control fibroblasts (*A*) and their released EVs (*B*). Lipid extracts from control and H-RasV12 cells and their released EVs were analysed by LC/MS-MS. Data are expressed as pg of lipid species/μg of proteins. Mean values ± S.D. (n = 9, cells; n = 6, EVs) are shown (*p<0.05, CTRL vs H-RasV12).
(PDF)

S5 Fig Detection of autotaxin associated to EVs. Samples were isolated from H-RasV12 expressing fibroblasts and cells transfected with the vector alone as control (CTRL). Cell extracts (30μg) and EV preparations (5μg) were separated by SDS-PAGE, electrotransferred and probed with anti-autotaxin antibody and with positive and negative markers commonly detected in EVs.
(PDF)

S6 Fig Neutral and acid sphingomyelinase activity in H-RasV12 and control fibroblasts. The activity of neutral (nSMase) and acid sphingomyelinase (aSMase) was measured using a fluorometric assay kit (Amplex Red Sphingomyelinase Assay Kit), in which SMase activity was directly proportional to the fluorescence emitted. Data are expressed as % of SMase activity in control samples (set 100). Mean values ±S.D. of three independent experiments.
(PDF)

S1 Table Number of lipid molecular species detected in control- and H-RasV12 expressing fibroblasts and their released EVs. PLaa, phospholipid acyl-acyl, PLae, phospholipid acyl-ether.
(TIF)

## References

[1] Mathivanan S, Ji H, Simpson RJ. Exosomes: extracellular organelles important in intercellular communication. J Proteomic.2010; 73: 1907–1920. 10.1016/j.jprot.2010.06.006 20601276

[2] Tetta C, Ghigo E, Silengo L, Deregibus MC, Camussi G. Extracellular vesicles as an emerging mechanism of cell-to-cell communication. Endocrine. 2013; 4411–4419. 10.1007/s12020-012-9839-0 PMC3726927 23203002

[3] Turturici G, Tinnirello R, Sconzo G, Geraci F. Extracellular membrane vesicles as a mechanism of cell-to-cell communication: advantages and disadvantages. Am J Physiol Cell Physiol. 2014; 306: 621–633. 10.1152/ajpcell.00228.2013 24452373

[4] Kowal J, Arras G, Colombo M, Jouve M, Morath JP, Primdal-Bengtson B, et al Proteomic comparison defines novel markers to characterize heterogeneous populations of extracellular vesicle subtypes. Proc Natl Acad Sci USA. 2016; 113: E968–977. doi: 10.1073/pnas.1521230113 26858453 10.1073/pnas.1521230113 PMC4776515

[5] Urbanelli L, Magini A, Buratta S, Brozzi A, Sagini K, Polchi A, et al Signaling pathways in exosomes biogenesis, secretion and fate. Genes. 2013; 4: 152–170. doi: 10.3390/genes4020152 24705158 10.3390/genes4020152 PMC3899971

[6] Urbanelli L, Buratta S, Sagini K, Ferrara G, Lanni M, Emiliani C. Exosome-based strategies for Diagnosis and Therapy. Recent Pat. CNS Drug Discov. 2015; 10: 10–27. 26133463 10.2174/1574889810666150702124059

[7] Record M, Poirot M, Silvente-Poirot S. Emerging concepts on the role of exosomes in lipid metabolic diseases, Biochimie. 2014; 96: 67–74. doi: 10.1016/j.biochi.2013.06.016 23827857 10.1016/j.biochi.2013.06.016

[8] Trajkovic K, Hsu C, Chiantia S, Rajendran L, Wenzel D, Wieland F, et al Ceramide triggers budding of exosome vesicles into multivesicular endosomes, Science. 2008; 319: 1244–1247. doi: 10.1126/science.1153124 18309083 10.1126/science.1153124

[9] Parolini I, Federici C, Raggi C, Lugini L, Palleschi S, De Milito A, et al Microenvironmental pH is a key factor for exosome traffic in tumor cells. J Biol Chem. 2009; 284: 34211–34222. doi: 10.1074/jbc.M109.041152 19801663 10.1074/jbc.M109.041152 PMC2797191

[10] Laulagnier K, Grand D, Dujardin A, Hamdi S, Vincent-Schneider H, Lankar D. JP, et al PLD2 is enriched on exosomes and its activity is correlated to the release of exosomes. FEBS Lett. 2004; 572: 11–14. doi: 10.1016/j.febslet.2004.06.082 15304316 10.1016/j.febslet.2004.06.082

[11] Subra C, Laulagnier K, Perret B, Record M. Exosome lipidomics unravels lipid sorting at the level of multivesicular bodies. Biochimie. 2007; 89: 205–212. doi: 10.1016/j.biochi.2006.10.014 17157973 10.1016/j.biochi.2006.10.014

[12] Carayon K, Chaoui K, Ronzier E, Lazar I, Bertrand-Michel J, Roques V, et al Proteolipidic composition of exosomes changes during reticulocyte maturation. J Biol Chem. 2011; 286: 34426–34439. doi: 10.1074/jbc.M111.257444 21828046 10.1074/jbc.M111.257444 PMC3190795

[13] Rappa G, Mercapide J, Anzanello F, Pope RM, Lorico A. Biochemical and biological characterization of exosomes containing prominin-1/CD133. Mol Cancer. 2013; 12: 62 doi: 10.1186/1476-4598-12-62 23767874 10.1186/1476-4598-12-62 PMC3698112

[14] Llorente A, Skotland T, Sylvänne T, Kauhanen D, Róg T, Orłowski A, et al Molecular lipidomics of exosomes released by PC-3 prostate cancer cells. Biochim Biophys Acta. 2013; 183: 1302–1309. 10.1016/j.bbalip.2013.04.011 24046871

[15] Lydic TA, Townsend S, Adda CG, Collins C, Mathivanan S, Reid GE. Rapid and comprehensive ‘shotgun’ lipidome profiling of colorectal cancer cell derived exosomes. Methods. 2015; 87: 83–95. doi: 10.1016/j.ymeth.2015.04.014 25907253 10.1016/j.ymeth.2015.04.014 PMC4615275

[16] Subra C, Grand D, Laulagnier K, Stella A, Lambeau G, Paillasse M, et al Exosomes account for vesicle-mediated transcellular transport of activatable phospholipases and prostaglandins. J Lipid Res. 2010; 51: 2105–2120. doi: 10.1194/jlr.M003657 20424270 10.1194/jlr.M003657 PMC2903822

[17] Urbanelli L, Magini A, Ercolani L, Sagini K, Polchi A, Tancini B, et al Oncogenic H-Ras up-regulates acid β-hexosaminidase by a mechanism dependent on the autophagy regulator TFEB. PLoS One 2014; 9: e89485 doi: 10.1371/journal.pone.0089485 24586816 10.1371/journal.pone.0089485 PMC3933543

[18] Malaquin N, Martinez A, Rodier F. Keeping the senescence secretome under control: Molecular reins on the senescence-associated secretory phenotype. Exp Gerontol. 2016; 82: 39–49. doi: 10.1016/j.exger.2016.05.010 27235851 10.1016/j.exger.2016.05.010

[19] Lehmann BD, Paine MS, Brooks AM, McCubrey JA, Renegar RH, Wang R, et al Senescence-associated exosome release from human prostate cancer cells. Cancer Res. 2008; 68: 7864–7871. doi: 10.1158/0008-5472.CAN-07-6538 18829542 10.1158/0008-5472.CAN-07-6538 PMC3845029

[20] Urbanelli L, Buratta S, Sagini K, Tancini B, Emiliani C. Extracellular Vesicles as New Players in Cellular Senescence. Int J Mol Sci. 2016; 17: 1408¸ doi: 10.3390/ijms17091408 27571072 10.3390/ijms17091408 PMC5037688

[21] Yu X, Harris SL, Levine AJ. The regulation of exosome secretion: a novel function of the p53 protein. Cancer Res. 2006; 66: 4795–4801. doi: 10.1158/0008-5472.CAN-05-4579 16651434 10.1158/0008-5472.CAN-05-4579

[22] Lespagnol A, Duflaut D, Beekman C, Blanc L, Fiucci G, Marine JC, et al Exosome secretion, including the DNA damage-induced p53-dependent secretory pathway, is severely compromised in TSAP6/Steap3-null mice. Cell Death Differ. 2008; 15: 1723–1733. doi: 10.1038/cdd.2008.104 18617898 10.1038/cdd.2008.104

[23] Brown W. Dynamic Light Scattering—The Method and some Applications (ed. Brown W.) 177 (Oxford Science Publications, 1993).

[24] Cermenati G, Giatti S, Audano M, Pesaresi M, Spezzano R, Caruso D, et al J Steroid Biochem Mol Biol. 2017; 168: 60–70. doi: 10.1016/j.jsbmb.2017.02.002 28167298 10.1016/j.jsbmb.2017.02.002

[25] Cermenati G, Audano M, Giatti S, Carozzi V, Porretta-Serapiglia C, Pettinato E, et al Lack of sterol regulatory element binding factor-1c imposes glial Fatty Acid utilization leading to peripheral neuropathy. Cell Metab. 2015; 21: 571–583. doi: 10.1016/j.cmet.2015.02.016 25817536 10.1016/j.cmet.2015.02.016

[26] Di Micco R, Fumagalli M, Cicalese A, Piccinin S, Gasparini P, Luise C, et al Oncogene-induced senescence is a DNA damage response triggered by DNA hyper-replication. Nature. 2006; 444: 638–642. doi: 10.1038/nature05327 17136094 10.1038/nature05327

[27] Armeni T, Ercolani L, Urbanelli L, Magini A, Magherini F, Pugnaloni A, et al Cellular redox imbalance and changes of protein S-glutathionylation patterns are associated with senescence induced by oncogenic H-ras. PLoS One 2012; 7: e52151 doi: 10.1371/journal.pone.0052151 23284910 10.1371/journal.pone.0052151 PMC3527427

[28] Krishnamurthy J, Torrice C, Ramsey MR, Kovalev GI, Al-Regaiey K, Sharpless NE. Ink4a/Arf expression is a biomarker of aging. J Clin Invest 114: 1299–1307 doi: 10.1172/JCI22475 15520862 10.1172/JCI22475 PMC524230

[29] Di Micco R, Sulli G, Dobreva M, Liontos M, Botrugno OA, Gargiulo G, et al Interplay between oncogene-induced DNA damage response and heterochromatin in senescence and cancer. Nat Cell Biol. 2011;13: 292–302. doi: 10.1038/ncb2170 21336312 10.1038/ncb2170 PMC3918344

[30] Becker T, Haferkamp S. Molecular Mechanisms of Cellular Senescence, in Wang Z, Inuzuka H, editors. Senescence and Senescence-Related Disorders. InTechOpen 2013, doi: 10.5772/56158

[31] King HW, Michael MZ, Gleadle JM. Hypoxic enhancement of exosome release by breast cancer cells. BMC Cancer. 2012; 12: 421; doi: 10.1186/1471-2407-12-421 22998595 10.1186/1471-2407-12-421 PMC3488584

[32] Lötvall J., Hill A.F., Hochberg F., Buzás E.I., Di Vizio D., Gardiner C., et al Minimal experimental requirements for definition of extracellular vesicles and their functions: a position statement from the International Society for Extracellular Vesicles. J Extracell Ves. 2014; 3: 26913; doi: 10.3402/jev.v3.26913 25536934 10.3402/jev.v3.26913 PMC4275645

[33] Pols MS, Klumperman J. Trafficking and function of the tetraspanin CD63. Exp Cell Res 2009; 315; 1584–1592. doi: 10.1016/j.yexcr.2008.09.020 18930046 10.1016/j.yexcr.2008.09.020

[34] Zöller M. Tetraspanins: push and pull in suppressing and promoting metastasis. Nat Rev Cancer. 2009; 9: 40–55. doi: 10.1038/nrc2543 19078974 10.1038/nrc2543

[35] Anliker B, Chun J. Lysophospholipid G protein-coupled receptors. J Biol Chem. 2004; 279: 20555–20558. doi: 10.1074/jbc.R400013200 15023998 10.1074/jbc.R400013200

[36] Hirsova P, Ibrahim SH, Krishnan A, Verma VK, Bronk SF, Werneburg NW, et al Lipid-induced signaling causes release of inflammatory extracellular vesicles from hepatocytes. Gastroenterol. 2016; 150: 956–967. 10.1053/j.gastro.2015.12.037 PMC4808464 26764184

[37] Jethwa SA, Leah EJ, Zhang Q, Bright NA, Oxley D, Bootman MD, et al Exosomes bind autotaxin and act as a physiological delivery mechanism to stimulate LPA receptor signalling in cells. J Cell Sci. 2016; 129: 3948–3957. doi: 10.1242/jcs.184424 27557622 10.1242/jcs.184424 PMC5087657

[38] Pienimaeki-Roemer A, Ruebsaamen K, Boettcher A, Orsó E, Scherer M, Liebisch G, et al Stored platelets alter glycerophospholipid and sphingolipid species, which are differentially transferred to newly released extracellular vesicles. Transfusion. 2013; 53: 612–626. doi: 10.1111/j.1537-2995.2012.03775.x 22804622 10.1111/j.1537-2995.2012.03775.x

[39] Pienimaeki-Roemer A, Kuhlmann K, Böttcher A, Konovalova T, Black A, Orsó E, et al Lipidomic and proteomic characterization of platelet extracellular vesicle subfractions from senescent platelets. Transfusion. 2015; 55: 507–521. doi: 10.1111/trf.12874 25332113 10.1111/trf.12874

[40] Wallner S, Schmitz G. Plasmalogens the neglected regulatory and scavenging lipid species. Chem Phys Lip. 2011; 164: 573–589. 10.1016/j.chemphyslip.2011.06.008 21723266

[41] Thai TP, Rodemer C, Jauch A, Hunziker A, Moser A, Gorgas K, et al Impaired membrane traffic in defective ether lipid biosynthesis. Hum Mol Genet. 2001; 10: 127–136. 11152660 10.1093/hmg/10.2.127

[42] Phuyal S, Skotland T, Hessvik NP, Simolin H, Øverbye A, Brech A, et al The ether lipid precursor hexadecylglycerol stimulates the release and changes the composition of exosomes derived from PC-3 cells. J Biol Chem. 2015; 290: 4225–4237. doi: 10.1074/jbc.M114.593962 25519911 10.1074/jbc.M114.593962 PMC4326831

[43] Hama H. Fatty acid 2-Hydroxylation in mammalian sphingolipid biology. Biochim Biophys Acta. 2010; 1801: 405–414. doi: 10.1016/j.bbalip.2009.12.004 20026285 10.1016/j.bbalip.2009.12.004 PMC2826524

[44] Guo BB, Bellingham SA, Hill AF. The neutral sphingomyelinase pathway regulates packaging of the prion protein into exosomes. J Biol Chem. 2015; 90: 3455–3467. 10.1074/jbc.M114.605253 PMC4319014 25505180

[45] Bianco F, Perrotta C, Novellino L, Francolini M, Riganti L, Menna E, et al Acid sphingomyelinase activity triggers microparticle release from glial cells. EMBO J. 2009; 28: 1043–1054. doi: 10.1038/emboj.2009.45 19300439 10.1038/emboj.2009.45 PMC2664656

[46] Ramírez de Molina A, Penalva V, Lucas L, Lacal J.C. Regulation of choline kinase activity by Ras proteins involves Ral-GDS and PI3K. Oncogene. 2002; 21: 937–946. doi: 10.1038/sj.onc.1205144 11840339 10.1038/sj.onc.1205144

[47] Lee S.J., Yi T., Ahn S.H., Lim D.K., Hong J.Y., Cho Y.K., Lim J., Song S.U., Kwon S.W.. Senescing human bone-marrow-derived clonal mesenchymal stem cells have altered lysophospholipid composition and functionality, J. Proteome Res. 13 (2014) 1438–1449. doi: 10.1021/pr400990k 24498988 10.1021/pr400990k

[48] Wang G, Dinkins M, He Q, Zhu G, Poirier C, Campbell A, et al Astrocytes secrete exosomes enriched with proapoptotic ceramide and prostate apoptosis response 4 (PAR-4): potential mechanism of apoptosis induction in Alzheimer disease (AD). J Biol Chem. 2012; 287: 21384–21395. doi: 10.1074/jbc.M112.340513 22532571 10.1074/jbc.M112.340513 PMC3375560

[49] Kakazu E, Mauer AS, Yin M, Malhi H. Hepatocytes release ceramide-enriched pro-inflammatory extracellular vesicles in an IRE1α-dependent manner. J Lip Res. 2016; 57: 233–245. 10.1194/jlr.M063412 PMC4727419 26621917

[50] Andreu Z, Yáñez-Mó M. Tetraspanins in extracellular vesicle formation and function. Front. Immunol. 2014; 5: 442 doi: 10.3389/fimmu.2014.00442 25278937 10.3389/fimmu.2014.00442 PMC4165315

