# Supplementary figures and images for: Extracellular vesicles released by fibroblasts undergoing H-Ras induced senescence show changes in lipid profile
Source: PLoS One. 2017 Nov 28;12(11):e0188840. doi: 10.1371/journal.pone.0188840 (PMC5705128; doi:10.1371/journal.pone.0188840)

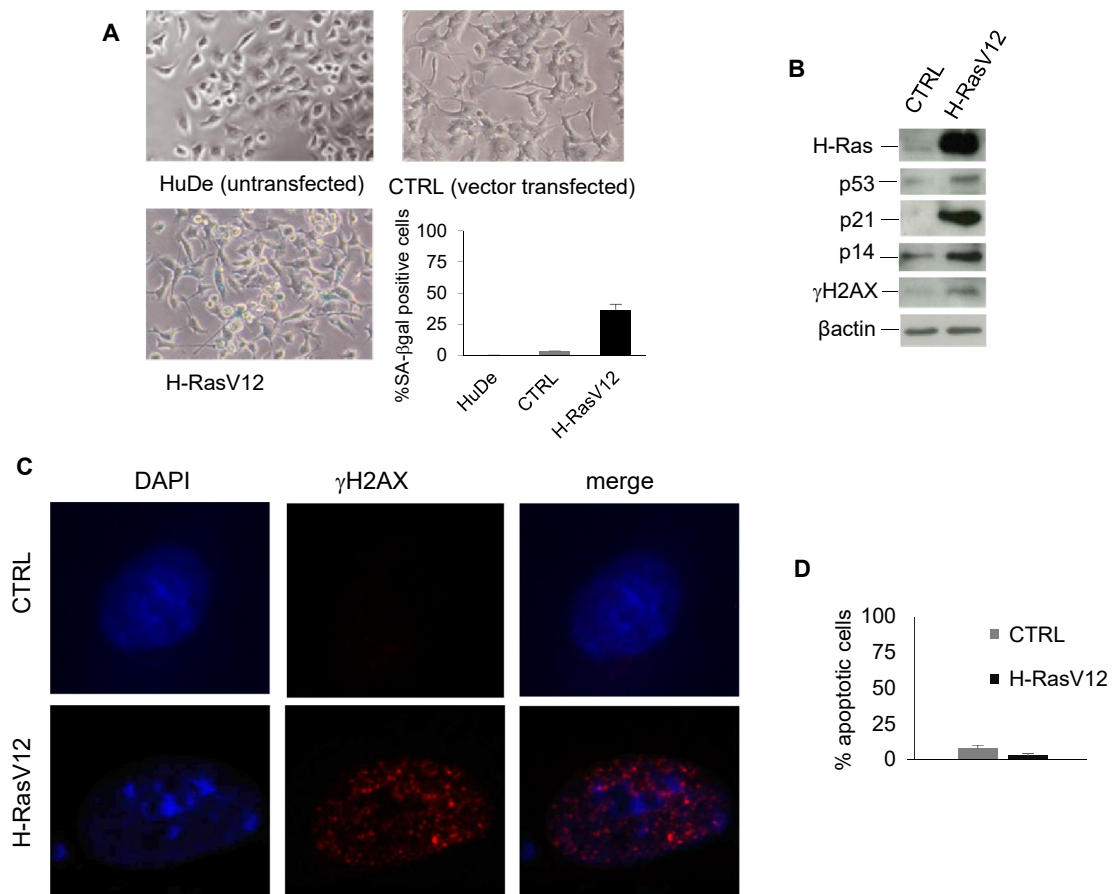

**S1 Figure**

Supplement: S1 Fig — A) Senescence-associated β-galactosidase staining. Microscopy images of HuDe (untransfected), CTRL (empty vector transfected) and H-RasV12 fibroblasts and quantification of SA-βgal positive cells. SA-β-gal positive cells were counted at least on three different fields in three independent experiments (*p<0.05, CTRL vs H-RasV12). B) Immunoblotting of OIS markers. Cell extracts (30 μg) were separated by SDS-PAGE, electrotransferred and probed with mouse monoclonal anti-p53, rabbit polyclonal anti-γH2AX, rabbit polyclonal anti-p14 ARF (Santa Cruz Biotechnology) and rabbit monoclonal anti-p21 (Cell Signaling Technology). C) Immunostaining for γH2AX. Cells were fixed in 4% paraformaldehyde, permeabilized with 0.1% Triton X-100 in PBS, incubated with an anti-γH2AX in 2% FBS/0.01% Triton X-100/PBS and labelled with an anti-rabbit Alexa-Fluor 594 antibody. Nuclei were stained with 1 μg/ml DAPI. Fluorescence microscopy analysis was carried out using a Nikon TE2000 microscope through a 60x oil immersion objective. D) Analysis of late apoptosis by detection of DNA content. Low molecular weight DNA produced by apoptosis-induced DNA fragmentation in CTRL and H-RasV12 fibroblasts was solubilized by cell permeabilization with 70% ethanol and high molecular weight DNA retained in fibroblasts quantified by DAPI staining. Cellular fluorescence was analysed by a NucleoCounter NC-3000 automated image analysis system (Chemometec) and the percentage of sub-G1 cells on total cells was quantified by NucleoView software. (PDF) [file pone.0188840.s001.pdf]

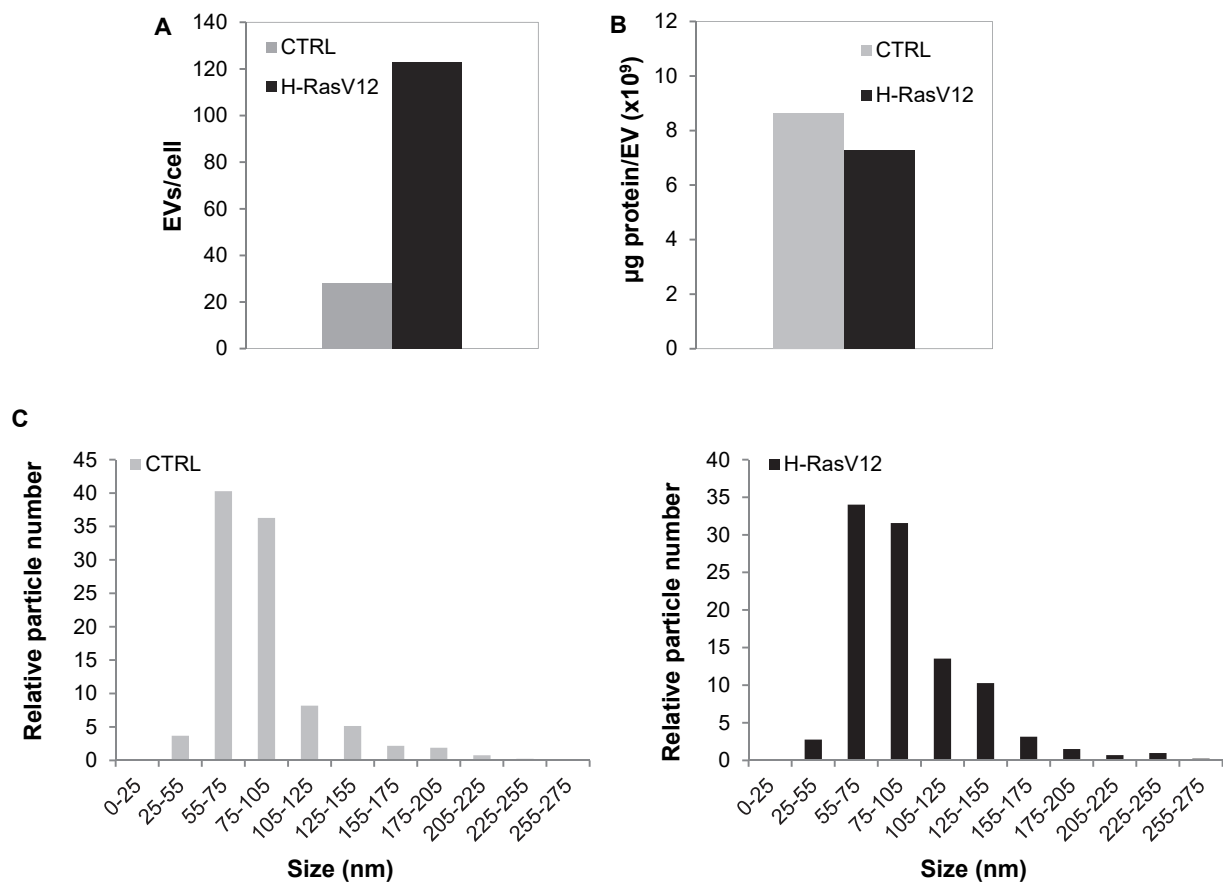

**S2 Figure**

Supplement: S2 Fig — A) Quantification of EVs. The amount of released EVs was measured by NTA and expressed as EVs/cell. Briefly, EVs pellets were resuspended in the amount of PBS (filtered through a 0.02 μm filter) needed to obtain a concentration within the recommended range (2 x 10^8^–1 x 10^9^ particles per ml) and vortexed for 1 min. Samples were then loaded into a NS500 instrument (Malvern, UK). Five videos, each of 60 s, were acquired for every sample and analysed by NTA 2.3 software. B) Determination of EVs protein content. The protein amount of EV preparations was quantified by Bradford assay and normalized for the amount of released EVs as measured by NTA, then expressed as μg protein/EV. The y-axis values are multiplied by 109. C) Representative particle size distribution of EVs from H-RasV12 fibroblasts (right panel) and control cells (left panel), reported as relative particle number, i.e. number of particles of the indicated diameter with respect to the total number of analyzed particles. For H-RasV12 we found a mean diameter of 104.0 ± 3.7 nm and mode of 76.5 ± 1.8 nm; for CTRL, we found a mean diameter of 95.1 ± 2.6 nm and mode of 77.9 ± 3.7 nm. (PDF) [file pone.0188840.s002.pdf]

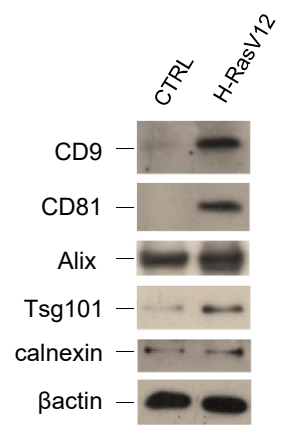

**S3 Figure**

Supplement: S3 Fig — EVs were isolated from H-RasV12 expressing fibroblasts and cells transfected with the vector alone as control (CTRL) by differential ultracentrifugation. Collected medium underwent centrifugation at 2000 x g for 10 min, then at 10,000 x g for 30 min and, finally, at 100,000 x g for 70 min. The final pellet was resuspended in PBS, centrifuged again at 100,000 x g for 70 min to wash it, then the total amount of pelleted proteins was determined by Bradford method. EV preparations (3μg) were separated by SDS-PAGE, electrotransferred and probed with the positive and negative markers indicated. (PDF) [file pone.0188840.s003.pdf]

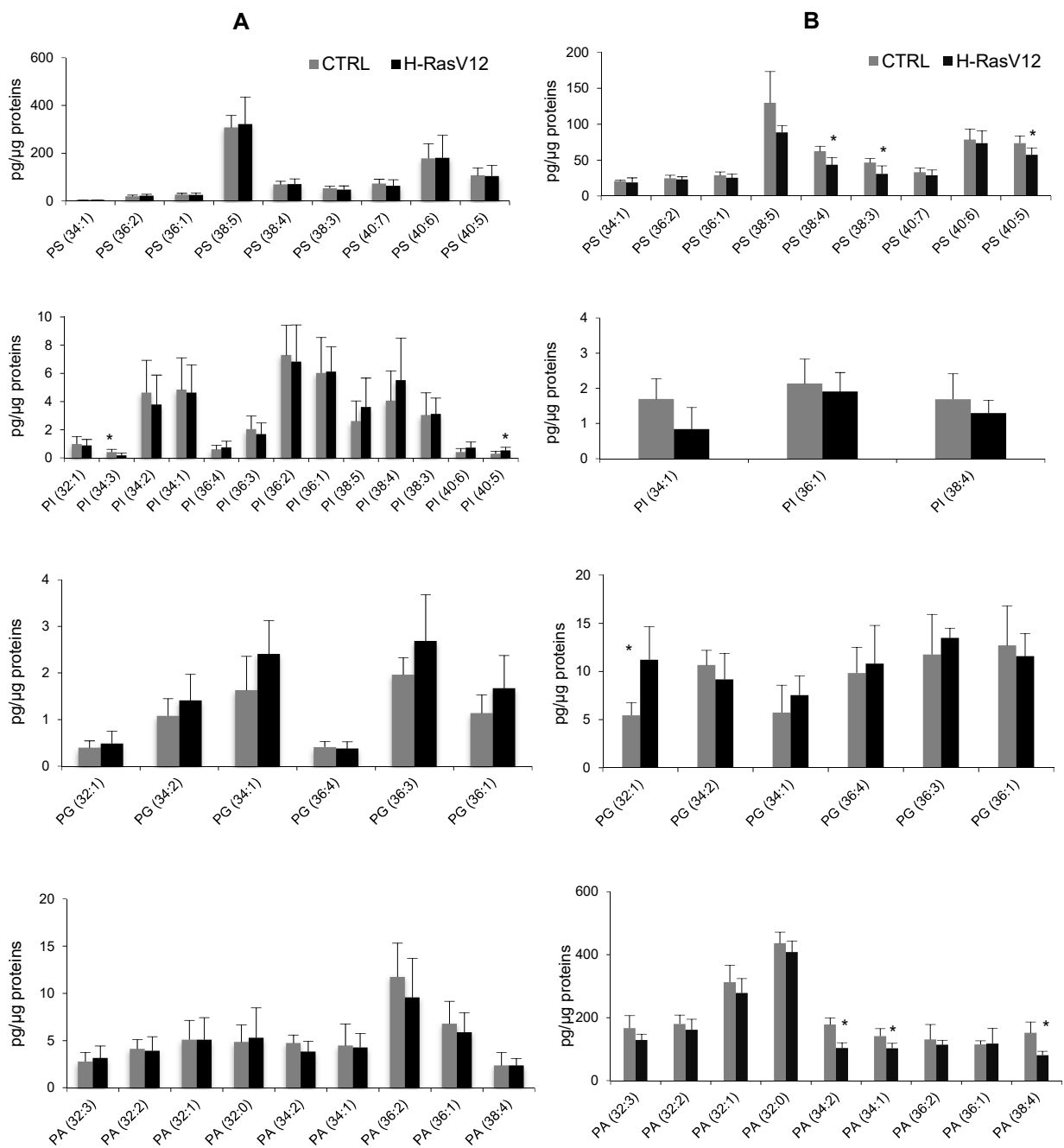

**S4 Figure**

Supplement: S4 Fig — Lipid extracts from control and H-RasV12 cells and their released EVs were analysed by LC/MS-MS. Data are expressed as pg of lipid species/μg of proteins. Mean values ± S.D. (n = 9, cells; n = 6, EVs) are shown (*p<0.05, CTRL vs H-RasV12). (PDF) [file pone.0188840.s004.pdf]

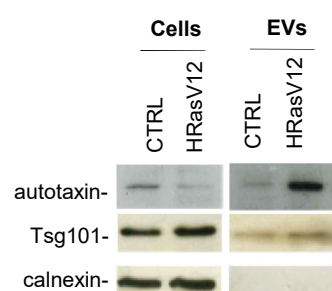

**S5 Figure**

Supplement: S5 Fig — Samples were isolated from H-RasV12 expressing fibroblasts and cells transfected with the vector alone as control (CTRL). Cell extracts (30μg) and EV preparations (5μg) were separated by SDS-PAGE, electrotransferred and probed with anti-autotaxin antibody and with positive and negative markers commonly detected in EVs. (PDF) [file pone.0188840.s005.pdf]

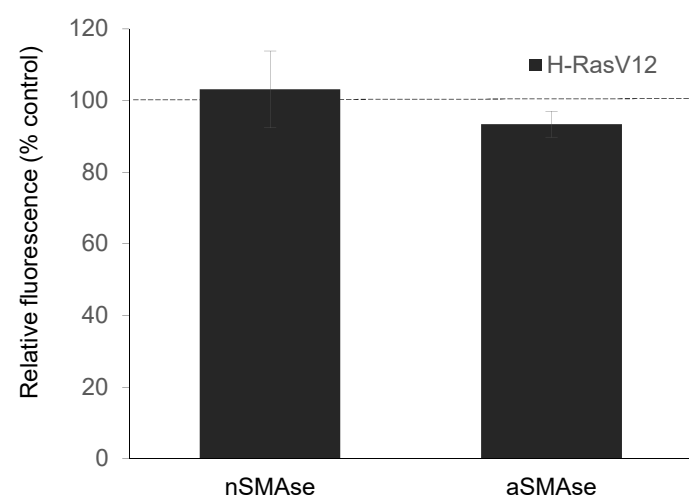

**S6 Figure**

Supplement: S6 Fig — The activity of neutral (nSMase) and acid sphingomyelinase (aSMase) was measured using a fluorometric assay kit (Amplex Red Sphingomyelinase Assay Kit), in which SMase activity was directly proportional to the fluorescence emitted. Data are expressed as % of SMase activity in control samples (set 100). Mean values ±S.D. of three independent experiments. (PDF) [file pone.0188840.s006.pdf]

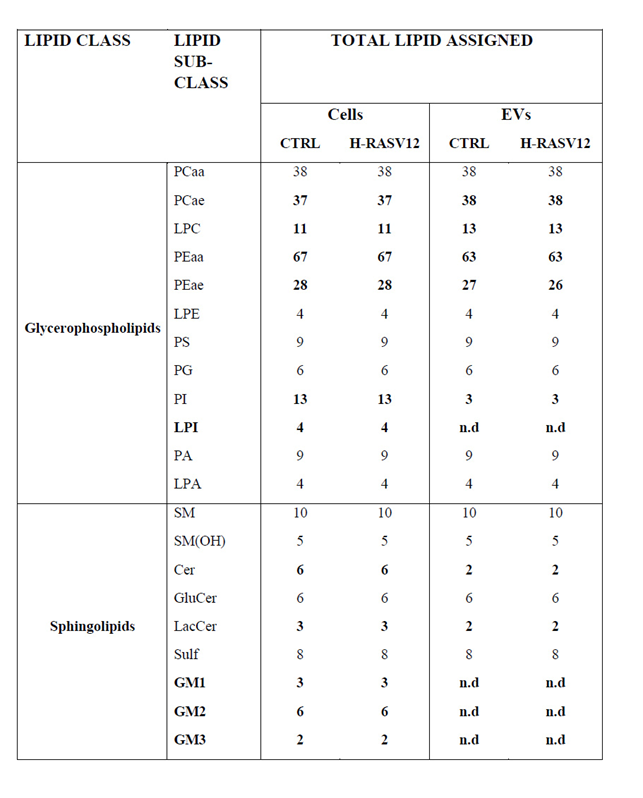

Supplement: S1 Table — PLaa, phospholipid acyl-acyl, PLae, phospholipid acyl-ether. (TIF) [file pone.0188840.s007.tif]
